# Clinical translation of injectable hydrogels: from bioactive polymers to long-acting drug delivery systems

**DOI:** 10.1007/s13346-025-02033-1

**Published:** 2026-01-22

**Authors:** Natalia Carballo-Pedrares, Virna Margarita Martín Giménez, María José Alonso

**Affiliations:** 1 Center for Research in Molecular Medicine and Chronic Diseases (CiMUS), University of Santiago de Compostela, Health Research Institute of Santiago de Compostela (IDIS), Santiago de Compostela, Spain; 2 Department of Pharmacy and Pharmaceutical Technology, School of Pharmacy, University of Santiago de Compostela, Santiago de Compostela, Spain; 3 Instituto Madrileño de Estudios Avanzados en Nanociencia (IMDEA Nanociencia), Madrid, Spain

**Keywords:** Biomaterials, Injectable hydrogels, Drug delivery systems, Clinical translation, Personalized medicine

## Abstract

Injectable hydrogels (IHs) have emerged as versatile biomaterials that enable localized therapy through minimally invasive delivery. Their in situ sol–gel transition supports sustained and targeted release of therapeutics, enhancing patient comfort and reducing dosing frequency. However, clinical translation remains limited due to challenges in achieving controlled degradation, ensuring long-term biocompatibility, scaling production, and meeting regulatory standards. Despite these hurdles, several IH-based formulations are progressing through clinical trials or have reached the market, underscoring their therapeutic potential. This review examines the major translational barriers and highlights recent advances that are accelerating the adoption of IHs in precision and personalized medicine.

## Introduction

Injectable hydrogels (IHs) are three-dimensional networks of hydrophilic polymers capable of retaining large amounts of water, designed to undergo in situ gelation upon injection and to support controlled delivery of therapeutics. IHs are gaining attention in a wide range of biomedical applications due to their feasibility for localised and minimally invasive treatments, thereby improving patient compliance [1]. Beyond essential biocompatibility, these polymeric networks may be engineered to exhibit controlled degradation and stimuli-responsive behaviour tailored to their target application [1]. Moreover, their ability to transition from sol to gel in situ allows for injection in liquid form and subsequent solidification at the target site, adapting to the tissue environment. Coupled with their capacity to encapsulate drugs or cells, IHs are well-suited for local drug delivery and tissue engineering in the context of various clinical applications [2].

The biomedical significance of hydrogels lies in their potential to bridge synthetic materials with biological systems. These systems mimic the extracellular matrix (ECM), providing an environment prone to cell adhesion, proliferation, and differentiation [3] and making them promising candidates for regenerative medicine. Clinically, IHs have been deployed in oncology, spinal function, and ophthalmic treatments, reflecting their versatility and therapeutic relevance.

Despite their potential, several challenges hinder the clinical translation of IHs. Although extensive research has enhanced the understanding of their properties, ensuring consistent performance and scalability remains difficult [1]. Material design must balance mechanical robustness, biodegradability, and biocompatibility, ensuring structural integrity under physiological conditions while degrading at a predictable rate without triggering immune responses [4]. Manufacturing processes must meet regulatory standards, emphasising the need for standardised protocols and early incorporation of critical quality attributes [5].

Bridging the gap from laboratory to innovation and large-scale production also presents some complexity. Preclinical studies frequently expose limitations in material performance, not evident in early-stage experiments [6]. Moreover, choosing appropriate animal models for preclinical studies is crucial to ensure reliability and translatability of research findings [7].

One recent success involves ionic fluid-based hydrogels, which undergo gelation upon injection and form localised depots for controlled drug release. Several variants of this strategy have shown efficiency in preclinical animal models and are advancing towards clinical trials [8].

This review explores hydrogel design considerations, identifies critical factors for clinical translation, and examines successful case studies to elucidate the elements required to bring these innovative technologies from bench to market.

A comprehensive literature search was conducted in PubMed, the FDA and EMA webpages and clinicalTrials.gov to identify publications, clinical trials and marketed products reporting on IHs with translational or clinical relevance. Search strings combined terms related to injectable hydrogels, drug delivery, biomaterials, and clinical translation using the following operators: (“injectable hydrogel” OR “ionogel”) AND (“clinical” OR “biomedical application” OR “drug delivery”).

Publications were included if they reported in vivo, preclinical, or clinical use of IHs for therapeutic. They were also included if described clinical translation, regulatory approval, or commercialisation of IH-based systems.

## Design and engineering of IHs

### The variety of biomaterials and resulting hydrogels

**Fig. 1:**
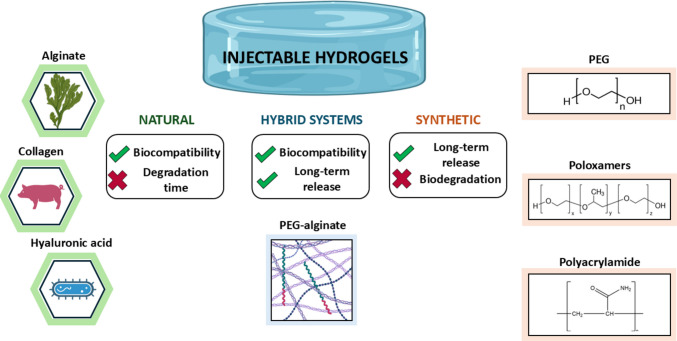
Schematic classification of IHs by polymer sources

IHs are composed of either natural or synthetic polymers, each offering unique advantages for biomedical applications (Fig. 1). Natural polymers such as alginate, chitosan, collagen, and hyaluronic acid (HA) have been widely attracted considerable attention due to their intrinsic biocompatibility and bioactivity. Since the 1980s, efforts have been made to bring these systems into clinical use, with early examples including collagen-based hydrogels for aesthetic applications such as Zyplast® and Fibrel®. Collagen, a primary component of the ECM, has been widely recognised for its ability to promote cell adhesion and proliferation [9]. Its early adoption was also motivated by its feasibility of extraction and excellent biocompatibility.

During the 1990s, HA- and cellulose-based hydrogels were introduced for the treatment of osteoarthritis and genitourinary disorders. These polymers offered enhanced versatility due to their functional groups, which allowed for chemical modifications to tailor hydrogel properties to specific therapeutic needs [9, 10]. More recently, in the early 2010s, focus shifted towards alginate- and chitosan-based hydrogels, further broadening the range of biopolymers used for clinical applications. Alginate is prized for its gentle gelation behaviour and capacity to encapsulate cells or drugs, making it highly suitable for tissue engineering and wound healing [11, 12]. One commercial example is Algisyl®, an alginate-based hydrogel used to treat heart failure by reinforcing the left ventricular wall.

Chitosan, on the other hand, stands out for its inherent antimicrobial properties, which make it a strong candidate for wound dressings and drug delivery systems [13, 14]. As in the case of BST-CarGel®, a chitosan-based hydrogel used since 2012 for osteoarthritis treatment. It supports cartilage repair when combined with bone marrow stimulation techniques, exemplifying the clinical value of biopolymer-based scaffolds.

Among synthetic polymers, PEG-based ones are the most extensively used for developing IHs. This polymer has led to marketed hydrogels in the early 2000s as PerioGlas®, an intraosseous gel designed to induce dental bone regeneration. PEG-based hydrogels are highly amenable to chemical modification, allowing control over crosslinking density. This tunability enhances their mechanical integrity and enables precise regulation of drug release profiles. An example is Jelmyto®, a PEG-based formulation used for intravesical delivery of Mitomycin in the treatment of urothelial cancer [15, 16].

Additionally, PEG derivatives such as Pluronic® block copolymers are widely employed due to their thermoresponsive behaviour, particularly useful in applications requiring sol–gel phase transitions under physiological conditions [17, 18]. However, the broader clinical application of PEG-based hydrogels has been limited by their poor in vivo degradability. Despite this, several devices are currently marketed for intra-articular use as bone fillers, including Dynagraft® and Actifuse®.

Polyacrylamide-based hydrogels also offer significant advantages, notably in their highly tunable mechanical properties. Their stiffness and elasticity can be precisely adjusted, making them suitable for a range of biomedical applications. The polymer's chemical structure supports functionalization and integration of bioactive agents. In clinical practice, polyacrylamide hydrogels such as NOLTREX® and Arthrosamid® have been used to treat osteoarthritis by providing mechanical support and pain relief [19, 20].

In recent years, hybrid and composite systems have emerged as a promising choice that combines the strengths of both natural and synthetic polymers. These hybrid hydrogels capitalise on the biological functionality of natural polymers while leveraging the mechanical robustness and tuneability of synthetic polymers [21–23]. Composites incorporating alginate and PEG, like Emdogain®, have demonstrated improved tissue regeneration when applied periodontally. PEG-fibrinogen hybrids are another example, employed to relieve joint pain in the treatment of early cartilage defects. Moreover, the integration of nanoparticles [24] or bioactive ceramics [25] into PEG-based matrices has further expanded their applicability in regenerative medicine and cancer treatment.

As medical devices, hydrogels used for genitourinary or osteoarthritic diseases are primarily evaluated for biocompatibility and physical performance. However, once they are engineered for drug delivery purposes, they fall under more complex regulatory pathways associated with pharmaceutical or combination products. This shift requires detailed pharmacokinetic data, stability evaluation, and rigorous clinical trials to ensure the safety and efficacy of the drug component. These regulatory demands highlight the importance of early-stage planning and interdisciplinary collaboration in the development of hydrogel-based drug delivery systems. Encouragingly, several systems currently in clinical trials are showing promising data for the controlled release of peptides and other bioactives.

Interestingly, ionic liquid-based hydrogels or ionogels represent an innovative class of in situ forming materials for localised drug delivery. These hydrogels incorporate organic salts with low melting points (ionic liquids, ILs) into polymeric networks, yielding materials with dual ionic and polymeric properties. Their unique features, including high thermal stability, ionic conductivity, low volatility, and exceptional solvation capability, make them advantageous for biomedical applications[8]. In injectable formulations, ILs promote rapid in situ gelation at the site of administration, forming structured drug depots that enhance the solubility of a broad range of bioactive agents (proteins, peptides, and small molecules). Additionally, ILs can exert multifunctional biological effects, such as antimicrobial activity, anti-inflammatory effects, and tissue repair [26].

Structurally, ionogels are composed of ILs either physically embedded in or covalently bonded to hydrogel matrices, typically based on natural polymers like oxidised hyaluronic acid, chitosan, or gelatin [27]. Their injectability is enhanced by their tuneable viscosity and intrinsic self-healing properties. Moreover, their electrical conductivity and responsiveness to magnetic stimuli open the door to integration with bioelectronic devices and advanced wound healing technologies [28].

### The behaviour of hydrogels as a function of the administration route

Various administration routes, such as subcutaneous, intramuscular, intratumoral, intra-articular and intraocular, have been explored to address specific clinical needs [29]. Below, we discuss the advantages, limitations and current perspectives associated with the most used routes for IHs administration.

#### Hydrogels administered by the subcutaneous route

Subcutaneous (SC) delivery is one of the most common methods for IHs, favoured for its minimally invasive nature and capacity to provide localised and sustained release of therapeutic agents. This route is particularly suitable for managing chronic disease, as it allows extended drug release, reducing dosing frequency and improving patient adherence [30]. A key advantage of SC-administered IHs is their ability to form depots at the injection site, which facilitates gradual drug diffusion into systemic circulation. This enhances the drug's half-life and minimises systemic side effects. Additionally, SC injections are typically easier to administer compared to other routes, making them suitable for outpatient care or even self-administration [31].

However, SC administration does present challenges. The local environment, including enzymatic activity and immune responses, can influence hydrogel degradation and drug release kinetics. Variables such as injection volume, viscosity, and mechanical properties must be optimised to avoid patient discomfort and inflammation. Achieving predictable biodegradation without triggering adverse immune reactions remains a major obstacle in these approaches [32]. Recent innovations include enzyme-responsive IHs, as those developed by Coulter et al. [33]. Based on a peptoid-peptide formulation covalently conjugated with zidovudine, this system showed sustained antiviral release over 35 days in an in vivo rat model by leveraging local phosphatase activity to trigger gelation [33].

Ionogels have been promising for SC delivery due to their structural adaptability and multifunctional nature. These systems integrate the ionic conductivity and stability of ILs with the robustness of polymer frameworks, offering precise control over drug release. Notable studies include IHs formed by PBAimBF4 and oxidised hyaluronic acid with antibacterial and fast-gelling properties, incorporating antibacterial imidazolium ILs for diabetic wound healing in a mouse model [34].

Talking about clinical translation, most of the hydrogels designed for subcutaneous use serve aesthetic purposes such as filling facial wrinkles or treating cutaneous scars (Table 6). It’s noteworthy to point out Vantas® and Supprelin LA®, marketed as drug delivery systems in the early 2000s for the treatment of prostate cancer and precocious puberty, respectively.

#### Hydrogels administered by the intramuscular route

Intramuscular (IM) injection is gaining attention in drug delivery, regenerative medicine, and vaccine development. The well-vascularized muscle tissue offers a suitable environment for drug absorption while maintaining drug reservoirs at the injection site [35].

Challenges associated with IM delivery include site discomfort, tissue irritation, and patient-specific variability, which can impact hydrogel behaviour. Since IHs are typically optimised for soft-tissue environments, the strong mechanical forces acting within muscles preclude depot stability and reproducible drug release profiles [35]. Furthermore, the IM route was targeted over the years for systemic absorption, reducing the clinical advantage of a bulky hydrogel system compared with simpler depot technologies (e.g. liposomes, microspheres).

Innovations such as needle-free jet injection systems, studied by Lawal et al., have shown promise in enhancing hydrogel penetration while minimising pain and tissue damage [36]. There remains a considerable way to go before an IM-targeted hydrogel can progress into clinical trials and ultimately reach the market. The last attempt was done in 2020 with GADinLADA®, an intrainguinal vaccine for the delivery of recombinant human glutamic acid dehydrogenase from an aluminium hydrogel (NCT04262479). The study was discontinued mostly due to administration site disorders, including muscle rupture.

#### Hydrogels administered by the intra-articular route

Intra-articular (IA) injection is an effective method for treating joint disorders, such as osteoarthritis (OA) and cartilage defects. This localised delivery targets the synovial cavity, offering sustained therapeutic presence with minimal systemic side effects [37–40].

One primary advantage of these systems is the ability to form a drug depot within the joint space, mitigating the rapid clearance typically seen with small-molecule drugs in synovial fluid. Furthermore, hydrogels can be engineered to mimic the viscoelasticity of the synovial environment, reducing joint friction and enhancing cushioning. These properties make IA hydrogels an outstanding alternative to surgical interventions [41, 42].

Similar to the IM route, the harsh mechanical environment within the joint can accelerate hydrogel degradation and compromise drug retention. Thereby, achieving long-term mechanical resilience and biocompatibility is a technical challenge. Disease progression heterogeneity among patients also requires tailored hydrogel formulations [43].

Recent advances have tackled these challenges. García-Couce et al. developed a thermosensitive chitosan/Pluronic® F127 hydrogel for IA delivery of etanercept [44] and dexamethasone [45], reducing inflammation and promoting cartilage regeneration in murine models. While Liu et al. evaluated a hydroxypropyl chitin/HA hydrogel that showed bio-lubricating and chondroprotective properties, suggesting dual functionality in joint lubrication and cartilage protection [39].

It is important to highlight that a wide range of IA-administered hydrogels are already commercialised for the treatment of articular defects, representing around 50% of all hydrogel-based systems on the market and nearly 40% of those currently in clinical trials. These include pain-relief products such as EUFLEXXA® and Orthovisc®, as well as bone fillers like PerioGlas® and Dynagraft®.

#### Hydrogels administered by the intratumoral route

Intratumoral (IT) administration of IHs represents an innovative strategy for localised cancer treatment. This approach allows direct injection into solid tumours, forming a localised drug depot that enhances retention and minimises undesired cytotoxic effects. IT hydrogels are under development for chemotherapeutics, immunotherapies and photothermal agents, enabling sustained and targeted drug exposure on the tumour site while reducing off-target effects [46].

Smart hydrogels can also respond to tumour-specific stimuli such as pH or enzymatic activity, enabling controlled drug release. Furthermore, for tumours that cannot be fully resected, IT hydrogels offer a minimally invasive therapeutic option [47–52].

However, the variability towards tumour environment, including variations in vascularisation and interstitial pressure, can hinder uniform hydrogel dispersion and drug penetration. Additionally, the immunosuppressive microenvironment and mechanical stress from injection can influence their therapeutic performance [53, 54].

Recent studies suggest that IL-based hydrogels could offer enhanced performance in this context. Certain ILs, particularly those with guanidinium cations and long alkyl chains, have demonstrated strong cytotoxic effects against various cancer cell lines, in some cases outperforming traditional chemotherapeutics like mitomycin C [55]. In this regard, it is noteworthy that a hydrogel formulation for mitomycin C release called Jelmyto® has already been commercialised. This system, based on a blend of Pluronic, PEG and HPMC, illustrates the feasibility of hydrogel-based chemotherapeutic delivery.

While IT hydrogels show strong potential, further research is needed to optimise their intratumoral diffusion. The development of stimuli-responsive hydrogels and combination therapies holds the potential to revolutionise cancer treatment through more precise therapeutic solutions.

#### Hydrogels administered by the intravitreal route

Intravitreal (IVT) delivery of IHs is an emerging strategy for treating retinal diseases, including age-related macular degeneration (AMD), diabetic retinopathy, and glaucoma. This approach enables direct drug delivery to the posterior segment of the eye, bypassing systemic circulation and ensuring localised, prolonged therapeutic action.

One key benefit of IVT hydrogel systems is their ability to extend drug residence time within the vitreous humour, reducing dose frequency and improving treatment adherence [56–61]. Hydrogels can encapsulate anti-angiogenic drugs, corticosteroids, or neuroprotective agents for controlled release over weeks or months. Additionally, IHs can support retinal tissue engineering by acting as scaffolds that support cell transplantation and modulate degenerative eye diseases [62–65].

Despite these advantages, the unique physicochemical environment of the vitreous humour poses challenges. Maintaining optical clarity is essential to avoid impairing vision, while ensuring controlled biodegradation without inflammation or toxicity remains a major hurdle. The small volume of the vitreous cavity further restricts the injectable volume, necessitating precise formulation adjustments to adapt the therapeutics [66]. Among all the cases reviewed, only one IVT hydrogel has been identified, which is derived from a decellularized human membrane for the treatment of retinal defects. The clinical trial is currently ongoing, with conclusive results expected over the course of the next year.

As shown in Table 1, each administration route should be selected based on therapeutic goals and drug features, always aiming to improve patient comfort and compliance [67].

**Table 1:** Comparison of administration routes for hydrogel delivery systems

Administration route	Injection site	Advantages	Challenges	Typical applications
Subcutaneous (SC)	Fatty layer under the skin	- Minimally invasive - Suitable for self-administration	- Limited volume (~ 1–2 mL) - Variable degradation rates	Chronic disease therapies (e.g. diabetes, hormonal therapy), monoclonal antibodies
Intramuscular (IM)	Muscle tissue	- Higher vascularisation leading to rapid systemic uptake - Larger volumes (~ up to 5 mL)	- More painful - Risk of nerve or blood vessel damage	Long acting injectables (e.g. vaccines)
Intra-articular (IA)	Joint space	- Reduces systemic toxicity - Hydrogels improve residence time in synovial fluid	- Technically demanding - Risk of infection or joint damage	Osteoarthritis, rheumatoid arthritis, and local anti-inflammatory therapies
Intratumoral (IT)	Tumoral tissue	- Maximises local drug concentration - Reduces systemic toxicity - Immune stimulation in situ	- Tumour accessibility hurdles - Heterogeneous distribution	Cancer immunotherapy, chemotherapeutic delivery, and localised gene therapy
Intravitreal (IVT)	Vitreous humour of the eye	- Direct delivery to the posterior eye - Minimises systemic exposure - Prolongs drug residence time	- Highly sensitive injection site - Risk of retinal detachment	Age-related macular degeneration, diabetic retinopathy, and ocular inflammation

Abbreviations: SC, subcutaneous; IM, intramuscular; IA, intra-articular; IT, intratumoral; ITV, intravitreal.

## Challenges in clinical translation

### Biocompatibility and safety concerns

Biocompatibility encompasses both short-term and long-term host responses, addressing the possible adverse biological responses triggered by a drug or medical device [68]. Ideally, materials should degrade in a controlled manner, producing non-toxic, metabolizable by-products [69]. Achieving this is challenging due to the diversity of hydrogel formulations, crosslinking mechanisms, and bioactive additives [70]. As discussed above, natural polymers typically excel in this regard due to their innate compatibility with biological pathways [71].

In vitro assessments, such as MTT assays, live/dead staining and transcriptomic profiling, provide insights into cytotoxicity and cellular stress responses [72]. However, in vivo evaluations using animal models are essential for understanding tissue integration, fibrosis and immune reactions. These are typically supported by imaging techniques like fluorescence microscopy or MRI, along with histological analyses [73].

Toxicity can arise from several sources, including the base polymer, crosslinking agents, degradation byproducts, and additives such as drugs or growth factors [74]. Traditional crosslinkers like glutaraldehyde are known for their cytotoxicity, prompting the development of safer alternatives like click chemistry and enzymatic or UV-activated methods [75].

Accumulation of acid by-products from PLGA degradation can lead to localised tissue irritation, inflammation and immune cell activation[76]. Designing mixed hydrogels with natural polymers often helps mitigate these risks [77].

Concerns also extend to other non-degradable polymers such as PEG with regard to its capacity to induce mild immune responses over time [78]. Indeed, multiple clinical reports and post-marketing studies have documented pre-existing anti-PEG IgG and IgM antibodies in 20–70% of human sera. These antibodies are thought to arise from prior environmental or pharmaceutical exposure (e.g., to PEGylated drugs, cosmetics, or food additives) [79]. For hydrogel systems employing PEG as a crosslinker or coating material, the immunologic risk depends on molecular weight, branching, and presentation. Covalently crosslinked PEG networks that do not release soluble PEG chains are generally less immunogenic than degradable or surface-exposed architectures that shed PEG fragments into circulation [80]. Regulatory guidance increasingly expects developers to evaluate anti-PEG antibody incidence and cross-reactivity when PEG is a major component of long-acting or systemic hydrogels. Preclinical screening can include in-vitro complement activation assays, binding ELISAs for anti-PEG IgG/IgM, and in-vivo monitoring for infusion-related reactions [81].

Conversely, while natural polymers (e.g., collagen, gelatin, alginate, chitosan, fibrin, hyaluronic acid) generally degrade into bioactive or easily cleared fragments, batch-to-batch variability can lead to inconsistent by-product profiles. Indeed, natural polymer hydrogels present a different immunogenicity profile, dominated by source-derived variability and residual contaminants. Because these materials are often harvested from animal or microbial origins, batch-to-batch differences in purity, molecular weight distribution, acetylation/deacetylation degree, and endotoxin burden can markedly influence local and systemic immune responses [78]. For instance, partially deacetylated chitosan has been reported to activate macrophages through Toll-like receptor pathways, however, this is not the case when chitosan is used in a highly purified form (medical grade). Similarly, alginates contaminated with polyphenols or endotoxins have been shown to elicit foreign body inflammation [82] and xenogeneic collagens containing residual telopeptides are known to trigger humoral immune recognition [83]. Even ostensibly “pure” hyaluronic acid preparations can induce transient inflammation if residual crosslinkers (e.g., BDDE) or protein impurities persist above specification limits [84]. Consequently, rigorous source control, depyrogenation, and chemical characterization are indispensable parts of a risk-based ISO 10993 program.

In this way, whereas for synthetic polymers, anti-PEG responses emphasize the need for immunological monitoring even for previously “inert” excipients, for natural polymers, biologic variability and residual contaminants highlight the importance of process validation and compositional analytics to assure reproducible host tolerance. In both cases, comprehensive ISO 10993-driven assessments (cytotoxicity, sensitization, systemic toxicity, and degradation product profiling) provide the empirical foundation to de-risk immune complications before first-in-human use.

For its part, hybrid materials provide tuneable properties but may generate unpredictable degradation profiles, especially when combined with inorganic additives like nanoparticles. These hybrids can raise long-term safety concerns related to bioaccumulation or interference with cellular signalling. As such, elucidating the metabolic and excretory pathways of all degradation products is imperative for clinical translation [4].

To aid risk-based planning of preclinical safety evaluations, Table 2 summarizes the biological evaluations for different types of hydrogels according to ISO 10993 standards, depending on their composition and route of administration.

**Table 2:** ISO 10993 biological evaluation matrix for IHs

Hydrogel — Route	Cytotoxic (10,993–5)	Irritative/ Sensitive (10,993–10)	Systemic toxicity (10,993–11)	Genotoxic (10,993–3)	Implants (10,993–6)	Hemocompatibility (10,993–4)	Chronic toxicity (10,993–11/3)	By-products (10,993–18)	Pyrogen/ Endotoxin
Alginate/ chitosan in situ forming hydrogels — SC/IM/IA	Yes	Conditional (SC route)	Yes	Conditional (if residual crosslinkers)	Conditional (IM/IA implants)	Conditional (if blood contact)	Conditional (if long-term implant > 30 days)	Yes (quantify enzymatic fragments)	Yes (critical for natural polymers)
Collagen/ fibrin hydrogels — SC/ IM	Yes	Yes	Yes	Conditional (if residual crosslinker)	Yes	Conditional (if blood contact)	Yes	Yes (quantify peptide fragments)	Yes (critical for natural polymer)
HA pre-formed gels — SC/IA	Yes	Yes	Yes	Conditional (if residual crosslinkers)	Conditional (IA implants)	Conditional (if blood contact)	Conditional (if long-term implant > 30 days)	Yes (quantify HA fragments or residual crosslinkers)	Yes (critical for natural polymer)
PEG-based hydrogels — IT	Yes	Yes	Yes	Yes (if acrylates)	Yes	Conditional (if blood contact)	Yes	Yes (quantify PEG fragments)	Yes
Thermoresponsive Pluronic gels— IT	Yes	Yes	Yes	No	Yes	Conditional (if blood contact)	Conditional (if long-term implant > 30 days)	Yes (residual solvents)	Yes
Acrylamide gels— IM	Yes	Yes	Yes	Yes (risk induced by acrylamide monomers)	Yes	Conditional (if blood contact)	Yes (long-term carcinogen)	Yes (quantify acrylamide monomers)	Yes
Bioceramics in hydrogel – IA/ bone filler	Yes (particle cytotoxicity conditional)	Yes	Yes	Conditional (if chemical additives)	Yes	Conditional (if blood contact)	Yes	Yes	Yes
Drug-loaded hydrogels— all routes	Yes (must include drug and matrix)	Yes	Yes	Yes	Conditional (IM/IA/IT implants)	Conditional (if blood contact or IV route)	Conditional (if long-term implant > 30 days)	Yes (quantify polymer and drug metabolite)	Yes
Ionogels—IT/SC	Yes	Yes	Yes	Yes (risk induced by ILs)	Conditional (IT implants)	Conditional (if blood contact)	Yes	Yes (quantify ILs metabolite)	Yes

Abbreviations: IT, intratumoral; SC, subcutaneous; IA, intra-articular; IM, intramuscular; IV, intravenous; HA, hyaluronic acid. The testing flow begins with the cytotoxicity assay (ISO 10993–5), which is the mandatory starting point, only after passing this test further evaluations are conducted in the following order: irritation and sensitization (10,993–10) → systemic toxicity (acute or subchronic, 10,993–11) → genotoxicity (10,993–3) → implantation and hemocompatibility (10,993–6 / 10,993–4) → chronic toxicity and degradation by-products (10,993–11 / 10,993–18) → pyrogenicity or endotoxin testing. “Conditional” label indicates that the test is required only in depicted cases.

### Assessment methods for biodegradation profiles

Biomaterial degradation is an inherently complex and multifactorial process involving physical, chemical, and mechanical changes that are often interdependent. Accurate evaluation of these processes is essential, particularly in biomedical applications where degradation profiles influence not only the functional lifespan of a material but also its biocompatibility and therapeutic performance [85]. Functional groups such as esters, ethers, amides, and anhydrides contribute to the degradation behaviour, leading to surface erosion, molecular weight changes, mass loss, and altered mechanical properties like tensile strength and viscosity [4].

Current methodologies for assessing degradation profiles can be broadly categorised into physical, mechanical, and chemical approaches. Among these, physical methods such as gravimetric analyses are widely used due to their simplicity. However, they often led to misinterpretation, as mass changes might be due to material solubility or water loss rather than real degradation [84]. Mechanical testing evaluates performance changes over time, offering insights into the structural integrity of the material. Still, they do not account for chemical breakdowns of biomaterials or by-products formed [79]. Chemical characterisation techniques, while more technically demanding, offer the most robust and regulatory-relevant assessment, as they allow for direct quantification of degradation products' compositional shifts.

Despite this, most hydrogel degradation studies still rely heavily on gravimetric analysis to report mass loss over time. While this offers a useful overview, it falls short of the stringent regulatory requirements, especially those defined by ISO 10993 standards, which emphasise comprehensive chemical profiling and toxicological risk assessment. Advanced analytical techniques such as Fourier-transform infrared spectroscopy (FTIR), high-performance liquid chromatography (HPLC), and mass spectrometry (MS) are thus essential for a rigorous understanding of hydrogels' biodegradation and for translation into clinical use.

### Biodegradation pathways

**Fig. 2:**
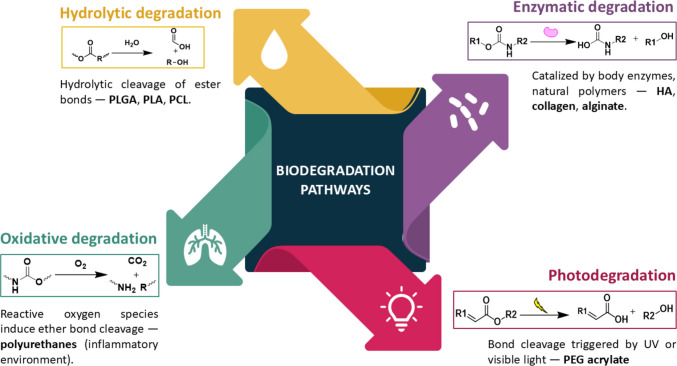
Representative biodegradation pathways for natural and synthetic polymers composing IHs

The chemical degradation of biomaterials used in IHs proceeds via distinct mechanisms, influenced by polymer structure, formulation, and surrounding physiological conditions. These degradation pathways, summarised in Fig. 2, are critical to the rational design of materials for specific biomedical applications.

#### Hydrolytic degradation

Synthetic polyesters such as poly (lactic-co-glycolic acid) (PLGA), polylactic acid (PLA), and polycaprolactone (PCL) undergo hydrolysis through cleavage of ester bonds. The resulting by-products, typically lactic and glycolic acid, are metabolised via natural pathways. Remarkably, PLGA degradation kinetics can be tuned by varying the lactic-to-glycolic acid ratio, with higher glycolic acid content accelerating hydrolysis [86–89].

Hydrolytic degradation, driven under physiological conditions, offers uniformity and reproducibility since it is not dependent on the enzymatic or metabolic activity of the patient. However, the resulting acidic microenvironment may trigger inflammatory responses, potentially leading to tissue irritation or immune reactions. This also accelerates localised breakdown, compromising mechanical integrity in load-bearing contexts. Therefore, strategies to buffer or neutralise acidic by-products are often considered to mitigate these potential drawbacks.

For its part, most ionogels are often designed with polycarbonate polymers, which can also degrade through hydrolysis under physiological conditions. However, IL components tend to persist longer, as many of them are not easily broken down within the body [90, 91]. Safe degradation of ionogels in the body requires careful design to ensure non-toxic and compatible with human metabolism byproducts [92, 93].

Because local depots of ionogels can result in prolonged tissue exposure and potential systemic distribution, developers should quantify parent and metabolite concentrations in plasma and organs using radiolabelled or LC–MS/MS approaches to define clearance pathways and margins of safety [94, 95]. Local effects such as inflammation, fibrosis, or delayed healing can occur even when in vitro cytotoxicity appears low, underscoring the need for subchronic implantation and histopathological evaluation consistent with ISO 10993–6/11. ILs may also modulate immune and complement pathways and can perturb blood components through surfactant-like mechanisms, warranting immunotoxicity and hemocompatibility testing [96]. Given the chemical diversity of ILs, genotoxicity assays are generally required, and long-term studies may be necessary for persistent formulations. Currently, no ionogel-specific regulatory guideline exists; agencies apply conventional ISO 10993 and ICH frameworks, emphasizing degradation profiling, ADME studies, and risk-based justification of the chosen ionic chemistry. Consequently, claims of “superior cytocompatibility” should be treated cautiously, as in vitro assays may underestimate chronic or systemic toxicity [97]. Comprehensive chemical characterization, metabolite identification, and in vivo distribution studies remain essential prerequisites for regulatory acceptance and clinical translation of ionogel systems.

#### Enzymatic degradation

Natural polymers such as collagen, alginate, and HA degrade through enzymatic cleavage catalysed by enzymes such as collagenase and hyaluronidase [98]. Notably, the degradation products can themselves be bioactive agents. For instance, HA oligosaccharides released during degradation have been proven to enhance tissue regeneration [99, 100]. Another example could be chitosan hydrogels, which are enzymatically cleaved by lysozymes and other enzymes, presenting antimicrobial activity [101].

However, enzymatic degradation is highly variable across patient populations, influenced by genetics, immune response, disease state and metabolic conditions. This makes standardisation and reproducibility particularly challenging, so most natural-based hydrogels are marketed as fillers instead of drug release systems.

#### Oxidative degradation

Synthetic polymers like polyurethanes are susceptible to oxidative degradation in the presence of reactive oxygen species (ROS), especially under inflammatory environments. This mechanism is particularly attractive for stimuli-responsive systems, as ROS levels are often elevated in cardiovascular pathologies such as atherosclerosis, hypertension, and ischemia–reperfusion injury [102]. By developing materials designed to degrade specifically in response to ROS, we can leverage this biological signal to trigger precise, localised material breakdown and subsequent drug release, ensuring targeted therapeutic effects. Materials designed to respond to oxidative cues offer spatial and temporal control over degradation. However, the unpredictability of oxidative stress levels and the potential toxicity of by-products remain important concerns [103].

#### Photodegradation

Photosensitive polymers offer another layer of control, undergoing degradation upon exposure to specific light wavelengths. For example, PEG-based hydrogels, such as PEG-acrylate, degrade upon UV or visible light exposure [104]. This precision is ideal for localised therapies, such as intratumoral delivery and ophthalmic treatments. However, photodegradable systems are limited by light penetration in tissues and potential instability under ambient light, restricting their broader clinical applicability [104].

### Manufacturing and scalability

Scalability and reproducibility are fundamental cornerstones in transitioning IHs from lab to clinic [1, 105]. This is particularly relevant in applications such as drug delivery, regenerative medicine, and tissue engineering, where batch consistency directly influences therapeutic efficacy and safety.

**Fig. 3:**
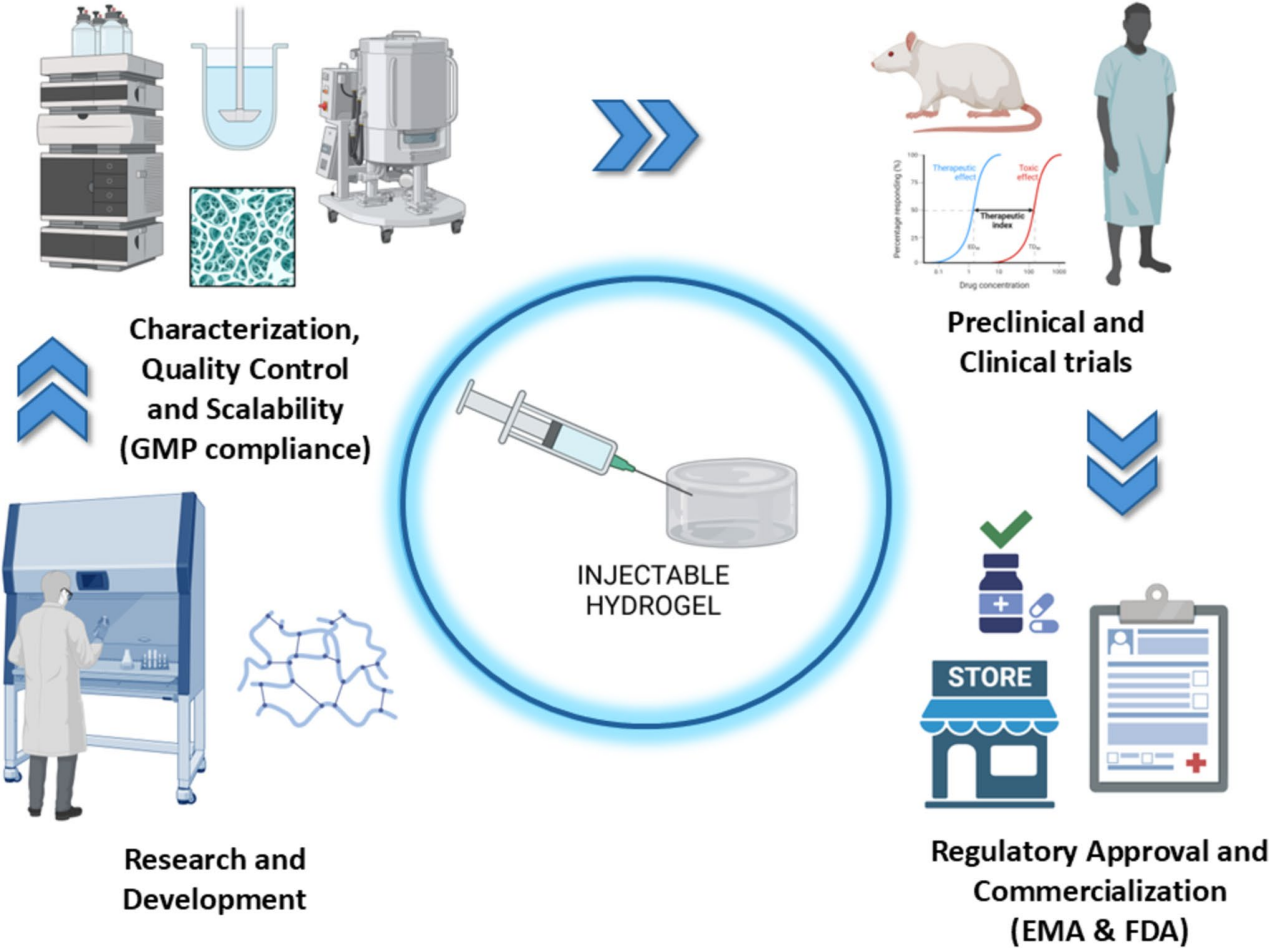
Representative scheme of the developing process from laboratory research to the commercialisation of an IH product

Reproducibility is often compromised by variability in raw materials, sensitivity to environmental conditions, and differences in manufacturing protocols [106]. A robust process must not only standardise formulation and crosslinking conditions but also ensure that degradation kinetics and biocompatibility remain within validated specifications. Figure 3 illustrates the stepwise approach required to transition from bench-scale development to clinically approved hydrogel products.

#### Raw materials and their standardisation

The core reproducibility in IHs systems is the consistent quality of raw materials. Most IHs are synthesised from polymers such as PEG, HA, or chitosan, typically functionalized with reactive groups to enable gelation. However, subtle variations in their molecular weight, functionalization degree, or purity can impact key hydrogel properties, including mechanical strength, gelation kinetics, and biocompatibility [107].

To address this, raw material sourcing and characterisation must be tightly standardised. Suppliers should provide detailed certificates of analysis, and materials should undergo rigorous in-house testing for critical parameters [108]. Without this level of control, downstream variability becomes almost inevitable, precluding reproducibility and regulatory compliance.

#### Manufacturing process control

Hydrogel production involves several independent steps, from polymer sterilisation to mixing it with crosslinkers or bioactives. Each stage introduces potential variability, underscoring the importance of rigorous process control [109].

Sterilisation is especially critical for clinical applications, yet it poses significant challenges. Techniques such as autoclaving, gamma irradiation, or filtration must be validated carefully, as they can alter polymer structure. For example, gamma irradiation, while effective, may induce chain scission in some polymers, affecting the mechanical properties of the final hydrogel [110]. Thus, formulation, sterilization and delivery device must be co-designed to preserve hydrogel performance while meeting sterility and usability requirements. Table 3 summarizes common polymer classes and their compatibility risks with standard sterilization modalities (chain scission, yellowing, residual toxicants), likely rheology drift and practical mitigations such as radical scavengers, lyophilized precursor kits and on-device mixing.

**Table 3:** Sterilization methods used for IHs

Polymer class	Sterilization methods	Compatibility risks	Rheology drift	Practical mitigations
Crosslinked HA or PEG hydrogels	Sterile filtration (common) EtO (common) Gamma radiation (rare)	Gamma radiation: chain scission	↓ viscosity / ↓ G' after ionizing radiation	Sterile filtration: when possible EtO cycle: long aeration Low radiation with antioxidants
Alginate-Ca^2^⁺ hydrogels	Sterile filtration (common) EtO (common) Gamma radiation (rare) Autoclave (for solutions)	Gamma radiation: chain scission Autoclave: alter hydrogels structure	↓ viscosity and altered gelation kinetics (weaker Ca^2^⁺ crosslinks)	Sterile filtration when possible EtO cycle: long aeration
Protein hydrogels (collagen, gelatine, fibrin)	Sterile filtration (common) EtO (common)	EtO accelerate proteolytic degradation	Denaturation, ↓ elasticity	Sterile filtration when possible EtO cycle: long aeration Minimize harsh heat
Acrylate hydrogels (PEG, PEGDA)	Sterile filtration (common) EtO (common) Gamma radiation (rare)	Photoinitiators may form reactive residues Gamma radiation: chain scission	↓ viscosity and altered gelation kinetics (reactive residues)	Sterile filtration when possible Use high-purity monomers Low radiation with antioxidants
Pluronic-based hydrogels	Sterile filtration (common) EtO (packaging)	EtO residues if not aerated	Rheology properties preserved	Sterile filtration when possible Minimize heat and cold cycles
Ionogels	Sterile filtration (common) EtO (packaging) Gamma radiation (rare)	EtO residues are problematic for lipophilic ILs Gamma radiation: chain scission	Not described	Sterile filtration at end point when possible
Polyurethane-based hydrogels	EtO (common) Gamma radiation (rare)	Gamma radiation: chain scission EtO residues Hydrolytic sensitivity	↓ G' after ionizing radiation	EtO cycle: long aeration Low radiation with antioxidants

Abbreviations: EtO, ethylene oxide; PEO, polyethylene oxide; PEG, poly(ethylene glycol); PEGDA, poly(ethylene glycol) diacrylate.

In parallel, successful clinical translation of IHs requires concurrent design of the formulation and its delivery device to ensure sterility, dose accuracy, usability, and regulatory compliance. Common configurations include pre-filled syringes, lyophilized reconstitution kits, and dual-barrel or on-mix cartridge systems, each offering distinct trade-offs in stability, user steps, and mixing efficiency [111]. Device materials must be compatible with the hydrogel formulation and chosen sterilization method, while extractables, leachables, and dead volume are carefully validated. Injection performance—expressed as peak plunger force versus injection rate for the intended syringe and needle—should be quantified early, as viscosity and gauge strongly influence human factors such as comfort and controllability. Designs should enable single-hand operation and minimize user error through tactile or visual cues. For high-viscosity gels, motorized or mechanically assisted applicators may be required [112]. Product stability strategy should be defined in parallel with device design: lyophilization, separated-component cartridges, or antioxidant-stabilized formulations can enable ambient storage, while cold-chain logistics remain necessary for protein- or cell-containing hydrogels. Packaging materials, barrier properties, and temperature excursion tolerances must be established through validated studies [113, 114].^.^ Integrating formulation, sterilization, and usability considerations from the outset facilitates manufacturability, regulatory acceptance, and consistent clinical performance.

Likewise, the mixing and crosslinking processes require strict regulation. Whether the crosslinking is physical, chemical, or enzymatic, it must occur under controlled conditions to ensure uniformity in hydrogel properties. Factors such as pH, temperature, and crosslinker concentration significantly influence the final product. Emerging technologies, like microfluidics and automated mixing systems, are increasingly being used to enhance process control, minimise operator errors, and improve reproducibility [115, 116].

#### Challenges in scaling up production

While laboratory-scale production allows for close control of conditions, scaling up introduces new challenges. Parameters that are easily managed in small volumes can become problematic at an industrial scale. Pilot-scale studies are essential to identify them early and adapt protocols accordingly [117]. Fortunately, advances in scalable microfluidic manufacturing maintain homogeneity during scale-up, making them attractive tools for translational research [118].

#### Quality control and regulatory compliance in scale-up processes

A robust quality control (QC) framework is indispensable when moving toward clinical or commercial application. QC must span the entire production chain, from raw materials to the final product, ensuring all the points discussed above, from batch-to-batch consistency to regulatory compliance.

Key QC parameters include gelation time, mechanical properties, swelling behaviour, degradation kinetics, and sterility [119]. A combination of analytical tools is typically employed, such as rheometry for viscoelastic profiling [120, 121], FTIR and NMR for chemical composition, and scanning electron microscopy (SEM) for microstructural analysis [122]. These assessments are especially relevant for applications such as tissue engineering, where mechanical cues influence cell behaviour and integration (Table 4).

**Table 4:** Quality parameters evaluated to assess the performance of IHs

CQA / CPP	Performance link	Measure method
Gelation time (37 °C)	Ensures in situ set within clinical window	Tube inversion, vial-in-cup rheology (temperature ramp) Gelation time: 30 s–5 min
Syringeability	Usability, determines required applicator	Dynamic injection test. Measure plunger force for the intended needle gauge Peak force < 200 N for single hand
Modulus window by route (G′)	Too soft → dispersion Too stiff → injection failure or tissue damage	Measure frequency sweep (1 Hz G′) at 37 °C. Use in vitro mechanical mimic (tissues) SC – 1 to 500 Pa IA – 100 to 2000 Pa IVT—< 50 Pa
Degradation half-life	Sets residence time, release duration and safety	In vitro mass loss In vivo implantation histology (ISO 10993–6) Quantify degradation by-products by LC–MS
Burst limit (initial release)	Prevents toxicity	In vitro release measure at 24 h (< 10–20% depending on the target)
Endotoxin and pyrogen	Safety for natural polymers	Raw natural polymers pyrogenation. Endotoxin quantification by batch (< 100 EU/mL)
Stability	Shelf-life and dosing accuracy	Measure viscosity and G′ modulus over time and after storage points
Residual solvent	Avoid toxicity risks	GC–MS / LC–MS
In vitro release mechanism	Translate to pharmacokinetic models (Korsmeyer–Peppas, Peppas–Sahlin or Hopfenberg)	Quantify drug release under multiple media conditions and relate it to rheological measures for storage modulus (G′) and swelling ratio Fit data into predictive modelling (report R^2^)

Abbreviations: IT, intratumoral; SC, subcutaneous; IA, intra-articular; IV, intravenous; IVT, intravitreal.

Swelling behaviour is also interesting since it influences drug release rates and structural stability. Standardised testing for swelling ratios and equilibrium water content is strongly recommended for consistent product performance [123].

Furthermore, sterility and microbiological testing are indispensable. Traditional culture-based sterility assessments may be complemented by more sensitive techniques such as PCR. Likewise, residual crosslinker levels should be evaluated using mass spectrometry or similar high-sensitivity methods to ensure long-term biocompatibility [124].

In conclusion, the successful scale-up of IHs requires meticulous attention to material consistency, process reliability, and rigorous QC protocols. Every step must align with regulatory expectations to ensure safety, efficacy, and functional integrity. The incorporation of advanced analytical tools and automation technologies offers a promising path forward, enhancing reproducibility and facilitating the clinical and commercial translation of these biomaterials.

## Clinical trials and approved products

A total of 81 injectable hydrogel-based systems were identified across clinical trials (30) and marketed products (51). These were classified by administration route, polymer family, product type and therapeutic indication. The IA route represented the largest fraction (45%), followed by SC (20%), IT (15%), IVT (10%), and others (e.g., intravesical). Natural polymers (e.g., HA, collagen, chitosan, alginate) accounted for 55% of systems, synthetic polymers (e.g., PEG, PVA, Pluronic®, polyacrylamide) for 35%, and composite systems for 10%. Among all products and trials, 87% were categorized as medical devices, 12% as pharmaceutical products, and 1% as cell-based therapies.

Approvals and new trial initiations increased steadily after 2010, with notable peaks in 2014–2016 (orthopedic fillers) and 2020–2023 (drug delivery systems, e.g., Jelmyto® and CartiLife®).

### Overview of ongoing clinical trials

The clinical trials summarised in Tables 5 and 6 provide an outline of the current progress and challenges in the translation of IH technologies from the laboratory to clinical application. These trials reflect a growing interest in exploiting the unique properties of hydrogels to address a wide range of medical needs, from tissue regeneration and drug delivery to innovative approaches in contraception.

**Table 5:** A non-exclusive list of IHs intended for drug delivery or cell therapy applications, ordered by year of the study start

Name	Composition	Therapeutic agent	Clinical purpose	Administration route	Clinical Trial ID/ year	State	Withdrawal reason	Quality
RMCL-CL001	Gelatin	Renal cells	Type II diabetes and chronic kidney disease	Kidney	NCT02525263/2016	Phase II	Therapy failed to improve renal function	Low
CLN-0046	PEG	Tyrosine kinase inhibitor	AMD	Intraocular	NCT03630315/2019	Phase I	Probable Phase II/III evaluation, no results report	Low
FX322	Pluronic	FX-332	Sensorineural hearing loss	Intratympanic	NCT04120116/2019	Phase II	Exploratory efficacy signals but limited clinical effect	High
Carti Life®	Fibrin	Autologous chondrocytes	Cartilage defects treatment	Intraarticular, knee	NCT05051332/2020	Phase III	No results report	Moderate
GADinLADA	Aluminium hydrogel	rhGAD65	Vaccination	Intrainguinal	NCT04262479/2020	Phase II	Safety proved but limited efficacy on C-peptide preservation	Low
Life Pearl®	PEG based microgel	Irinotecan	Colorectal cancer	Intraarterial	NCT04595266/2021	Phase II	No results report	High
Cart Revive®	Dextran-HA derivatives	-	Cartilage defects treatment	Intraarticular, knee	NCT05186935/2022	Phase II	No results report	Moderate
Re Space™	PEG	-	Post radiotherapy, spacer	Transperineal	NCT05369221/2022	Phase I	Probable Phase II/III evaluation, no results report	Low
UGN-102	PEG-PPO triblock copolymer	Mitomycin	Bladder cancer	Intravesicular	NCT05243550/2022	Phase III	Clinical efficacy proved, would require further confirmation	High
TumoCure®	Bulk polymer	Cisplatin	Head and neck cancer	Intratumoral	NCT05200650/2023	Phase I	No results report/Recruiting	Low
Colorectal cancer treatment	Pluronic® F68 and F127	Fluorouracil	Colorectal cancer	Intrarectal	NCT06385418/2024	Phase II	No results report/Recruiting	Moderate

Abbreviations: AMD, age-related macular degeneration; ECM, extracellular matrix; HA, hyaluronic acid; MI, myocardial infarction; PEG, polyethylene glycol; PPO, poly (p-phenylene oxide); PVA, polyvinyl alcohol; rhGAD65: recombinant human glutamic acid dehydrogenase; NCT, ClinicalTrials.gov identifier. The status of all clinical trials is Completed, unless otherwise specified in the column Withdrawal reason (active, recruiting).

**Table 6:** A non-exclusive list of IHs undergoing clinical trials as medical devices, ordered by year of the study start

Name	Composition	Clinical purpose	Administration route	Clinical Trial ID/year	State	Withdrawal reason	Quality
Bulkamid®	Collagen	Female stress urinary incontinence	Transurethral	NCT00629083/2008	NA	Comparison to Contigen showed no significative efficacy differences	High
ESS505	Polyethylene terephthalate	Contraception	Intravasal	NCT01664052/2021	NA	Removed from the market on 2018 due to declining sales	High
Gut Guarding Gel	Alginate – calcium salt	Gastrointestinal tumour	Intratumoral	NCT03321396/2017	NA	Safety proved, no Phase II/III studies performed	Low
PAAG-OA	Polyacrylamide	Treatment of pain in osteoarthritis	Intraarticular, knee	NCT04179552/2019	NA	No results report	High
PROMGEL-OA	HPMC	Treatment of pain in osteoarthritis	Intraarticular, knee	NCT04061733/2019	NA	No results report	Low
PVA Hydrogel	PVA	Treatment of pain in osteoarthritis	Intraarticular, knee	NCT04693104/2019	NA	No results report	Moderate
BP-009	Unknown polymeric hydrogel	Post radiotherapy, spacer	Transperineal	NCT05423431/2020	NA	No results report	Low
Controtide®	Polynucleotide	Meniscus lesion	Intraarticular	NCT05322005/2020	NA	Comparison to Hyalubrix showed no significative efficacy differences	Moderate
Embozene®	Polyzene F-microspheres	Treatment of pain in osteoarthritis	Intraarticular, knee	NCT04379700/2020	NA	Safety proved, probable Phase II/III evaluation	Moderate
NOLTREX™	Polyacrylamide with silver ions	Arthrosis pain relief	Intraarticular, knee	NCT06429319/2020	NA	Therapeutic efficacy was not enough consistent	Moderate
SMI-01	Silk particles in hydrogel carrier	Tissue filler	Subcutaneous	NCT04534660/2020	NA	No results report	Moderate
JointRep®	Chitosan	Cartilage defects treatment	Intraarticular, knee	NCT04840147/2021	NA	No results report/Recruiting	High
OptiSphere®	Microspheres	Treatment of pain in osteoarthritis	Intraarticular, knee	NCT04951479/2021	NA	Therapeutic efficacy was not enough consistent	Low
ADAM ™	Styrene maleic acid	Contraception	Intravasal	NCT05134428/2022	NA	No result report/Active	Moderate
Plenhyage®	Polynucleotide	Scars treatment	Intradermal	NCT05239117/2022	NA	No result report	Moderate
ArToFILL	HA hydrogel	Treatment of pain in osteoarthritis	Intra-articular	NCT06422169/2024	NA	No result report/Recruiting	Moderate
Human membrane hydrogel	Decellularized human membrane	Retinal defect treatment	Intraocular	NCT06433284/2025	NA	No result report/Active	Moderate
Hydrafil™	Unknown hydrogel	Degenerative disc disease	Intradiscal	NCT06011551/2025	NA	No result report/Recruiting	High

Abbreviations: AMD, age-related macular degeneration; ECM, extracellular matrix; HA, hyaluronic acid; MI, myocardial infarction; NA, Not Applicable is used to describe trials without FDA-defined phases, including trials of devices or behavioural interventions; HPMC, hydroxypropyl methylcellulose; NCT, ClinicalTrials.gov identifier. The status of all clinical trials is Completed, unless otherwise specified in the column *Withdrawal reason* (active, recruiting).

A distinction was made between Table 5 and Table 6 to reflect the different regulatory frameworks relevant to clinical translation. Hydrogels described in Table 5 are intended for drug delivery or cell therapy applications and therefore classified under pharmaceutical regulations as conventional drugs. By contrast, the hydrogels listed in Table 5 fall under the category of medical devices, for which pharmacokinetic evaluation is not required; instead, regulatory approval primarily depends on evidence of biocompatibility and therapeutic performance.

Methodological quality appraisal was evaluated within the following standards: low (exploratory, Phase 1, single-arm, non-randomised, small sample size > 20 patients), moderate (randomised, single-arm studies with adequate sample size 20–50 patients) and high (randomised, controlled, multicentre, adequate sample size > 50 patients).

#### Hydrogel design tendencies observed in clinical trials

IHs are currently being explored across a broad range of therapeutic areas, including cartilage repair (CartRevive®, *NCT05186935*) and cancer treatment (Life Pearl®, *NCT04595266*). Thanks to their biomimetic properties and ability to deliver therapeutic agents in a localized and sustained manner, IHs have emerged as strong candidates not only in regenerative medicine but also in oncology.

As shown in Tables 5 and 6, ongoing clinical trials feature hydrogels derived from both natural (e.g., alginate, HA) and synthetic polymers (e.g., polyacrylamide, PEG). This diversity allows for tailored control over critical characteristics, such as mechanical strength and degradation rates, making it possible to adapt formulations for different clinical needs. That said, despite the growing interest in hybrid systems, no current trials include mixed composite formulations. This absence underscores the persistent challenges in translating more complex hydrogel systems from preclinical research into clinical studies.

A recurring focus across many trials is site-specific drug delivery, aimed at maximizing treatment efficacy while minimizing systemic side effects. For instance, intratumoral hydrogels like *Gut Guarding Gel* (*NCT03321396*) provide localized drug delivery directly at tumour sites from an alginate matrix, thereby reducing the adverse effects associated with systemic administration.

#### The most significant factors to discontinue trials

**Fig. 4:**
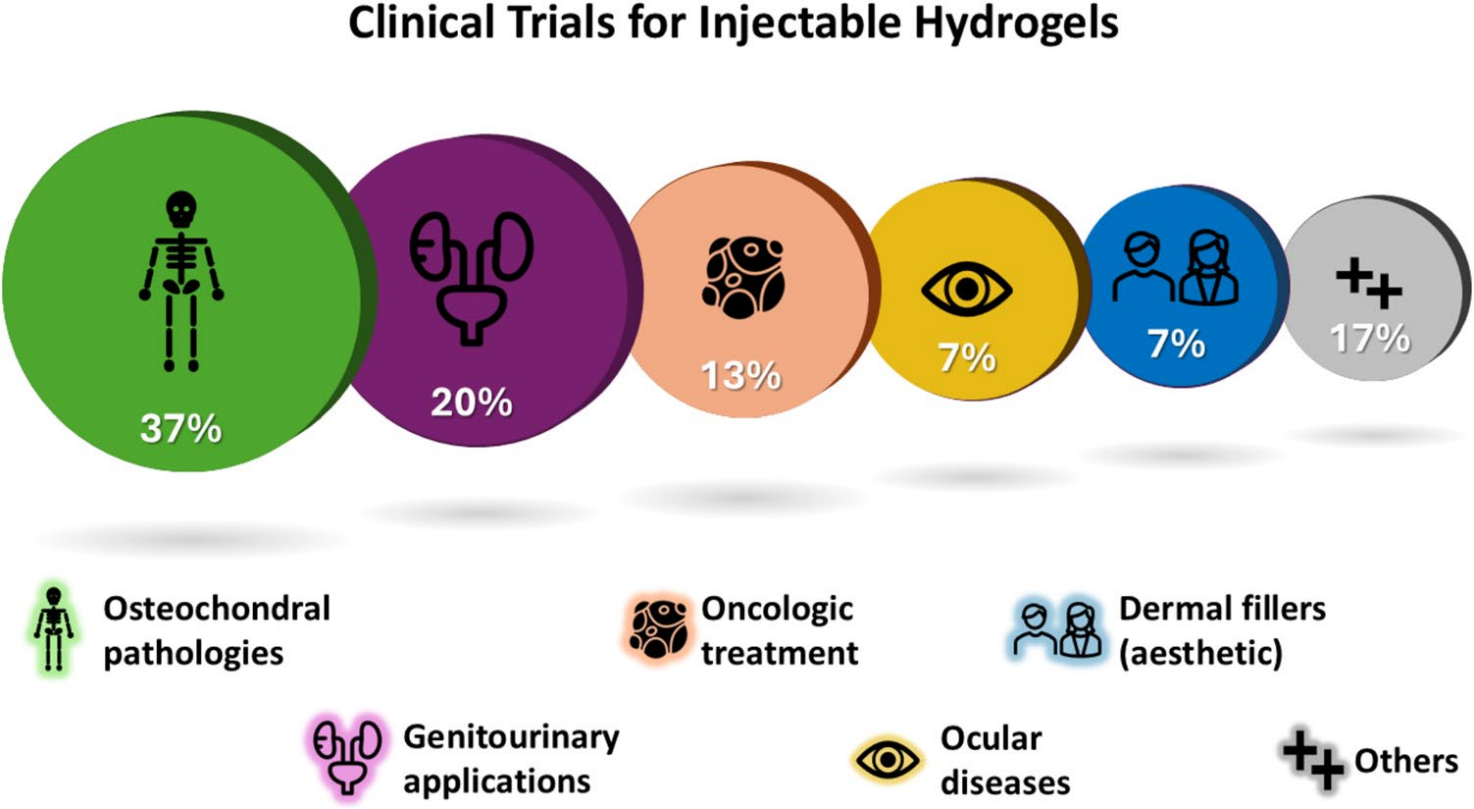

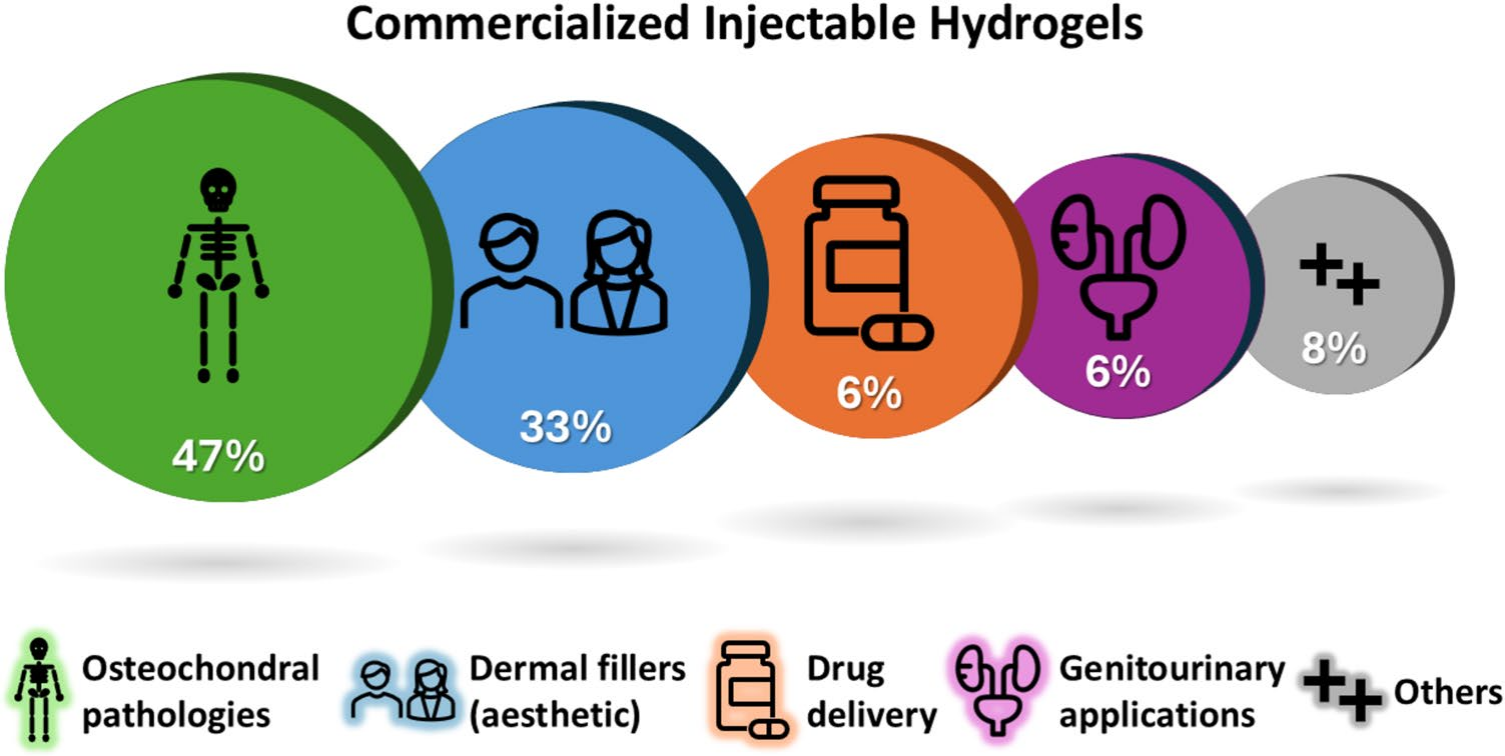
Overview of IHs products classified by their clinical purposes

The range of clinical trials currently underway (Fig. 4) highlights the transformative potential of IHs across diverse fields of medicine. However, the gap between what is observed in clinical trials and what ultimately reaches the market remains significant. According to FDA regulations, clinical results from Phase I or II studies are not required to be reported if they are inconclusive, making it difficult to ascertain the reason behind the withdrawal of many drug prototypes.

Based on the data compiled from these processes and clinical trials, summarised in Tables 5 and 6, we hypothesize that the primary reasons for failure can be categorized into three key factors.

The most significant factor reported include the lack of efficacy. Even when preclinical results are promising, some hydrogels simply do not show meaningful clinical benefits in human trials. For example, Noltrex™, a polyacrylamide hydrogel designed for osteoarthritis pain relief (NCT06429319), was ultimately discontinued due to insufficient efficacy consistency. In some cases, market comparison becomes a decisive factor, as seen with Controtide® (NCT05322005) and Bulkamid® (NCT00629083), which demonstrated clinical efficacy but did not show any significant advantages over existing marketed alternatives.

Inadequate study designs also plays a critical role. Trials with poorly defined endpoints, small sample sizes, and a lack of randomization can lead to inconclusive or misleading results as for ReSpace™ (NCT05369221) or Gut Guarding Gel (NCT03321396) which completed safety assessment but faced challenges to move further into efficacy evaluation.

Lastly, financial and logistical constraints are difficult to overcome. For example, the development of ESS505, a polyethylene-based hydrogel intended for contraceptive use (NCT01664052), was discontinued due to declining commercial performance and the emergence of competitive long-acting reversible contraceptive options.

As discussed above, another key hurdle is regulatory, while inert hydrogels used in applications such as joint or dermal fillers are typically classified as medical devices, drug-loaded hydrogels fall under pharmaceutical regulations. This distinction subjects them to far more rigorous approval pathways under agencies such as the FDA or EMA. Thus, even though the safety and usability of hydrogels have been well-established in biomedical applications, the added complexity of drug delivery requirements significantly narrows the field of viable candidates for commercial translation. Table 7 presents a comparative overview of the FDA (U.S.) and EMA (Europe) approval pathways, highlighting the principal regulatory requirements for the transition from clinical research to market access.

**Table 7:** Comparison between FDA and EMA regulatory pathways

Aspect	FDA (U.S.)	EMA (Europe)
Application Pathways	NDA (New Drug Application) BLA (Biologic License Application) PMA (Premarket Approval)	MAA (Marketing Authorization Application)
Clinical phases	Phase 1: 20–100 healthy volunteers (1 year) Phase 2: 100–300 patients (2 years) Phase 3: 1,000–3,000 patients (3–5 years)	
Review process	Single agency review (FDA)	Centralized via EMA National competent authorities may review
Advisory committee review	Required for complex applications	Committee for Medicinal Products for Human Use
Approval Scope	U.S. market only	All EU member states (via centralized procedure)
Post-Approval Monitoring	Phase IV studies Pharmacovigilance report	Risk management plans Post-marketing studies Environmental impact reports
Emergency Use Authorization (EUA)	Granted during public health emergencies for unapproved products or unapproved uses of commercialized products	Conditional Marketing Authorization (CMA) can be granted during public health emergencies (e.g., COVID-19 vaccines)
Orphan Drug Designation	For rare diseases (< 200,000 patients) Incentives: 7-year market exclusivity, tax credits, FDA fee waivers	For rare diseases (< 5 in 10,000 people) Incentives: 10-year market exclusivity, research grants, protocol assistance
Drugs and Medical Devices classification	Drugs: Prescription vs OTC Medical Devices: Class I (low risk) Class II (moderate risk, often requires 510(k)) Class III (high risk, requires PMA)	Drugs: risk considered in approval type Medical Devices: Class I (low risk) Class IIa/IIb (medium risk) Class III (high risk, requires conformity assessment)

Abbreviations: 510 (k), FDA regulatory process used for medical devices to demonstrate a substantially equivalency to another device already legally marketed; OTC, drugs without prescription required; PMA, pre-market approval.

Borderline cases, such as cell-laden hydrogels, often raise regulatory ambiguity regarding their classification as Advanced Therapy Medicinal Products (ATMPs). ATMPs, as defined by the EMA, encompass gene therapy, somatic-cell therapy, and tissue-engineered products that use cells, genes, or engineered tissues to achieve a therapeutic effect. Cell-laden hydrogels such as RMCL-CL001 (NCT02525263) or CartiLife® (NCT05051332) combine living cells with biomaterial scaffolds, bridging between medical devices and biological medicines. Their classification depends on factors as the primary mode of action, the degree of cell manipulation, and whether the therapeutic outcome is driven mainly by the biological activity of the cells or by the physical properties of the scaffold.

### Approved hydrogel-based drug delivery systems

Tables 8, 9, 10 offer a detailed overview of IHs that have been brought for both medical and aesthetic use. These products support a variety of clinical applications, from tissue regeneration to dermal fillers or cancer treatments. Together, they provide a comprehensive insight into the diverse technological advancements in biomaterials, helping to understand their applications and potential benefits in everyday clinical practice. These tables also include a classification in terms of hydrogel type, within three categories, depending on whether they are already-formed hydrogels injected as a ready-made viscoelastic implant (pre-formed viscoelastic gel), materials delivered in a liquid or precursor form and polymerizes/sets at the site (in situ gelation), or hydrogels used primarily as a temporary carrier or vehicle for particles / biologics (composite/carrier).

**Table 8:** Non-exclusive list of commercialised IH products for aesthetic use, following intradermal administration ordered by year of approval

Product name	Composition	Year/Authority of approval	Clinical purpose	Hydrogel type
Zyplast(R)® Zyderm(R)®	Collagen from animal source	1981/FDA (USA)	Correction of contour deficiencies	Pre-formed viscoelastic gel
Fibrel®	Collagen	1988/EMA (EU)	Cutaneous scars	In situ gelation
Sculptra®	PLA	2004/FDA (USA)	Facial wrinkles	Composite (carrier)
Hylaform®	Modified HA from avian source	2004/FDA (USA)	Facial wrinkles	Pre-formed viscoelastic gel
Elevess®	HA/ Lidocaine	2007/FDA (USA)	Moderate to severe facial wrinkles and folds	Pre-formed viscoelastic gel
Evolence® CollagenFiller	Collagen	2008/FDA (USA)	Facial wrinkles	Pre-formed viscoelastic gel
Ellansé®	HPMC/ PCL microspheres	2009/EMA (EU)	Facial wrinkles	Composite (carrier)
Juvéderm® XC	HA/ Lidocaine	2010/FDA (USA)	Facial wrinkles	Pre-formed viscoelastic gel
Belotero balance®	HA	2011/FDA (USA)	Facial wrinkles	Pre-formed viscoelastic gel
Bellafill	Collagen with PMMA microspheres/ Lidocaine	2015/FDA (USA)	Long-lasting dermal filler for acne scars	Composite (carrier)
Radiesse®	Hydroxyapatite and HPMC/ Lidocaine	2015/FDA (USA)	Correction of wrinkles, stimulation of natural collagen production	Composite (carrier)
Restylane® Lyft, Restylane® Refyne, Restylane® Defyne	HA/ Lidocaine	2016/FDA (USA)	Facial wrinkles	Pre-formed viscoelastic gel
Teosyal® RHA	HA	2017/FDA (USA)	Facial wrinkles and folds	Pre-formed viscoelastic gel
Revanesse® Versa +	HA/Lidocaine	2018/FDA (USA)	Facial wrinkles and folds	Pre-formed viscoelastic gel
Revanesse® Lips +	HA	2020/FDA (USA)	Filler-looking lip	Pre-formed viscoelastic gel
RHA® 2, 3, 4, Redensity	HA and BDDE/ Lidocaine	2021/FDA (USA)	Perioral rhytids	Pre-formed viscoelastic gel
Skinvive by Juvéderm	HA	2023/FDA (USA)	Facial wrinkles	Pre-formed viscoelastic gel

Abbreviations: BDDE, 1,4-butanediol diglycidylether; HA, hyaluronic acid; HPMC, hydroxypropyl methylcellulose; PCL, polycaprolactone; PLA, polylactic acid; PMMA, polymethylmethacrylate; FDA, U.S. Food and Drug Administration; PMA – Premarket Approval; 510(k) – Premarket Notification (U.S. device clearance pathway); SSED, Summary of Safety and Effectiveness Data.

**Table 9:** Commercialised IH products for orthopaedic use ordered by year of approval

Product name	Composition	Year/Authority of approval	Administration route	Clinical purpose	Hydrogel type
Emdogain®	Porcine decellularized matrix and PEG-alginate	1996/FDA (PMA, USA)	Flapless injection	Regenerates periodontal tissue	Composite (carrier)
Synvisc Hylan G-F 20	HA derivatives	1997/FDA (PMA, USA)	Intraarticular, knee	Treatment of pain in osteoarthritis	Pre-formed viscoelastic gel
Osteogenic protein 1(OP-1®) implant, OP-1® Putty	Collagen and HPMC/ Recombinant OP-1	2001/FDA (HDE, USA)	Spinal	Posterolateral lumbar spinal fusion	Composite (carrier)
Infuse® bone graft	Collagen/ BMP2	2002/FDA (PMA, USA)	Spinal	Spinal fusion and orthopaedic trauma surgeries	Composite (carrier)
EUFLEXXA®	HA	2004/FDA (PMA, USA)	Intraarticular	Treatment of pain in osteoarthritis	Pre-formed viscoelastic gel
Orthovisc®	HA	2004/FDA (PMA/ USA)	Intraarticular	Treatment of pain in osteoarthritis	Pre-formed viscoelastic gel
Coaptite®	HPMC salt and hydroxyapatite	2005/FDA (PMA, USA)	Submucosal	Female stress urinary incontinence	Composite (carrier)
PerioGlas®	PEG and glycerine/ Calcium phosphosilicate particles	2005/FDA (510(k), USA)	Intraosseous	Dental bone regeneration	Composite (carrier)
Dynagraft II®	DBM in Pluronic	2005/FDA (510(k), USA)	Intraosseous	Bone void filler	Composite (carrier)
Optium DBM Gel®	DBM in glycerol	2005/FDA (510(k), USA)	Spinal	Bone graft extender and void filler	Composite (carrier)
Grafton DBM gel®	DBM in glycerol	2005/FDA (510(k), USA)	Spinal	Bone graft extender and void filler	Composite (carrier)
AlloFuse Plus Paste®, AlloFuse Plus Putty®	Allographic DBM in PEG-PPO	2011/FDA (510(k), USA)	Spinal	Void filler, graft extender	Composite (carrier)
Gel-One®	Cinnamic acid functionalized HA	2011/FDA (PMA, USA)	Intraarticular	Treatment of pain in osteoarthritis	Pre-formed viscoelastic gel
Solesta®	HA/ Dextranomer	2011/FDA (PMA, USA)	Rectal	Fecal incontinence	Composite (carrier)
BST-CarGel®	Chitosan	2012/EMA (EU), Canada	Intraarticular	Cartilage repair	In situ gelation
Kinex Bioactive Gel®	Collagen and HA	2013/FDA (USA)	Intraosseous	Bone void filler	Composite (carrier)
TraceIT® Hydrogel Tissue Marker	PEG	2013/FDA (USA)	Percutaneous	Improved soft tissue alignment for image-guided therapy	In situ gelation
Arthrosamid®	Polyacrylamide	2014/EMA (EU)	Intraarticular	Treatment of pain in osteoarthritis	Pre-formed viscoelastic gel
Algisyl-LVR® Hydrogel Implant	Alginate	2014/EMA (EU)	Percutaneous	Advanced heart failure	In situ gelation
Monovisc®	High molecular weight HA crosslinked with biscarbodiimide	2014/FDA (USA)	Intraarticular	Treatment of pain in osteoarthritis	Pre-formed viscoelastic gel
SINOVIAL®	HA	2014/EMA (EU)	Intraarticular	Treatment of pain in osteoarthritis	Pre-formed viscoelastic gel
SpaceOAR®	PEG	2015/FDA (USA)	Transperineal, percutaneous	Prostate cancer radiotherapy	In situ gelation
Hymovis®	HA 500–730 kDa, functionalized with hexadecylamine	2015/FDA (USA)	Intraarticular	Treatment of pain in osteoarthritis	Pre-formed viscoelastic gel
Cingal®	High molecular weight HA crosslinked with biscarbodiimide/ Triamcinolone hexacetonide	2016/EMA (EU)	Intraarticular	Treatment of pain in osteoarthritis	Pre-formed viscoelastic gel
GelrinC®	PEG diacrylate and denatured fibrinogen	2017/EMA (EU)	Intraarticular	Treatment of focal cartilage lesions	In situ gelation
TRIVISC®	HA	2017/EMA (EU)	Intraarticular	Treatment of pain in osteoarthritis	Pre-formed viscoelastic gel
Durolane	HA	2017/FDA (USA)	Intraarticular, knee	Treatment of pain in osteoarthritis	Pre-formed viscoelastic gel
SYNOJOYNT®	HA salt	2018/FDA (USA)	Intraarticular	Treatment of pain in osteoarthritis	Pre-formed viscoelastic gel
Actifuse®	Pluronic® F127 and silica phosphate salt	2018/EMA (EU)	Intraosseous	Bone void filler in spinal and orthopaedic application	Composite (carrier)
Tactoset®	HA and calcium phosphate	2019/FDA (USA)	Intraosseous	Bone void filler for orthopaedic application	In situ gelation
Bulkamid®	Polyacrylamide	2020/FDA (USA)	Transurethral	Female stress urinary incontinence	Pre-formed viscoelastic gel

Abbreviations: BMP2, bone morphogenetic protein-2; DMB, demineralized bone matrix; HA, hyaluronic acid; HPMC, hydroxypropyl methylcellulose; PEG, polyethylene glycol; PPO: poly (p-phenylene oxide); HDE, Humanitarian Device Exemptin; FDA, U.S. Food and Drug Administration; PMA – Premarket Approval; 510(k) – Premarket Notification (U.S. device clearance pathway); SSED, Summary of Safety and Effectiveness Data.

**Table 10:** Commercialised IH products for drug delivery ordered by year of approval

Product name	Composition	Year/Authority of approval	Administration route	Clinical purpose	Hydrogel type
Vantas®	pHEMA and HPMA/ Histrelin acetate and Gonadotropin hormone	2005/FDA (USA)	Subcutaneous	Palliative treatment of prostate cancer	Composite (carrier)
Supprelin LA®	pHEMA/Histrelin acetate	2007/FDA (USA)	Subcutaneous	Central precocious puberty	Composite (carrier)
Jelmyto®	Pluronic® F127, PEG 400, HPMC/ Mitomycin	2020/FDA (USA)	Catheter instillation	Upper tract urothelial cancer	In situ gelation

Abbreviations: HPMA, poly (2-hydroxypropyl methacrylate); HPMC, hydroxypropyl methylcellulose; PEG, polyethylene glycol; pHEMA, poly (2-hydroxyethyl methacrylate); FDA, U.S. Food and Drug Administration.

#### Technological advancements and material innovations

Advances in biomaterials such as HA, collagen, PLA, and demineralized bone matrix (DBM) have significantly improved the biocompatibility, safety, and overall effectiveness of IHs. Innovations in crosslinking methods, controlled-release systems, and bioactive polymer design have extended product lifespan and enhanced therapeutic performance. These improvements have made IHs increasingly appealing across both medical and aesthetic fields, supporting broader clinical adoption.

#### Clinical efficacy and safety

Products that demonstrate clear therapeutic benefits with minimal adverse effects tend to see the most success in clinical settings. For example, orthopaedic and regenerative therapies like OP-1®, Infuse® Bone Graft, and Cingal® have shown effective tissue regeneration and osteoarthritis pain management, supporting their continued use in clinical practice.

Similarly, drug delivery hydrogels such as Vantas®, Supprelin LA®, and Jelmyto® have proven effective in treating a range of pathological conditions. These systems enhance drug stability, reduce dosing frequency, and improve patient compliance, all while maintaining well-established safety profiles.

#### Patient acceptance and minimally invasive procedures

Minimally invasive, non-surgical options have become increasingly popular among both patients and healthcare providers, primarily due to reduced recovery times and lower procedural risks. IH-based treatments for osteoarthritis, such as Synvisc®, Durolane®, SpaceOAR®, and Orthovisc®, offer effective symptom relief without the need for a surgical intervention, contributing to treatment adherence.

In the realm of drug delivery, products like Vantas® and Supprelin LA® provide long-acting effects via simple subcutaneous injection, reducing dose frequency. Meanwhile, Jelmyto® enables targeted chemotherapy *through* catheter instillation, increasing local drug retention while minimizing systemic exposure and side effects. These features not only improve therapeutic outcomes but also make such treatments more acceptable and practical for patients in routine care.

In summary, the success of the products outlined in the table is the result of a combination of scientific advancements, regulatory compliance, patient preferences, and market dynamics.

## Future directions and innovations

It has been made clear that the main limitations of IHs in clinical translation stem from biocompatibility, mechanical stability, and regulatory challenges. Many hydrogels face issues in balancing their residence time on the body, as rapid degradation can compromise their therapeutic effect, while a slower one may lead to bioaccumulation and toxicity. Sterilization and reproducibility are also major concerns, as most polymers may degrade or lose functionality when exposed to common sterilization techniques. Furthermore, regulatory approval requires extensive preclinical and clinical studies to ensure safety and efficacy, which can be time and cost-consuming. Hence, overcoming these limitations is crucial for the successful clinical application of IHs.

There are several aspects that prove the study of IHs to be a truly promising field with a bright future. For instance, advances in 3D printing and biofabrication are revolutionizing the design of IHs, enabling precise customization of their structure and composition for specific applications. 3D printing allows for the creation of intricate hydrogel networks that mimic native tissue architectures, enhancing their applicability in regenerative medicine and drug delivery [114]. Moreover, the development of smart hydrogels with stimuli-responsive release mechanisms holds great promise for targeted and controlled therapeutic delivery, minimizing side effects and improving efficacy [115].

Likewise, personalized medicine is driving the demand for patient-specific hydrogel systems, where biomaterials can be tailored based on individual clinical conditions, genetic profiles, and therapeutic goals. By incorporating patient-derived cells or biomolecules into hydrogels, these systems can be fine-tuned to address unique physiological environments, enhancing therapeutic outcomes [116].

Additionally, the future of IHs lies in their integration as advanced diagnostic tools, such as biosensors and imaging technologies. Hydrogels embedded with diagnostic markers or sensors can provide real-time feedback on therapeutic efficacy, disease progression, or biomarker levels. This combination of therapy and diagnostics could enable more dynamic and responsive treatment regimens, paving the way for precision medicine applications [117].

Therefore, the progress of IH technology relies heavily on interdisciplinary collaboration between materials science, biology, and clinical medicine. Materials scientists contribute innovative polymer designs, biologists offer insights into cellular interactions, and clinicians ensure these systems meet practical therapeutic needs. These advancements have the potential to reshape drug delivery, regenerative medicine, and patient care in unprecedented ways.

## Conclusion

IHs have emerged as versatile biomaterials, with expanding applications in drug delivery, tissue engineering, and regenerative medicine. Their unique properties, such as biocompatibility, tuneable degradation, and stimuli-responsive behaviour, position them as transformative tools in modern healthcare.

Despite these advancements, bringing IHs from the lab to the clinic remains a challenge. Issues like scalability, reproducibility, regulatory compliance, and the need to manage immune responses and potential toxicity continue to pose significant barriers to translational research.

Looking ahead, the field is moving towards next-generation technologies, including 3D printing, smart hydrogels, and patient-oriented designs. When combined with advanced diagnostic methods, these approaches could significantly transform the landscape of hydrogel-based therapies. By addressing current limitations and encouraging cross-disciplinary collaboration, IHs are well-positioned to make a huge impact on personalized medicine, enhancing patient outcomes and redefining therapeutic strategies across a wide range of clinical applications.

## References

[1] Almawash S, Osman SK, Mustafa G, El Hamd MA. Current and future prospective of injectable hydrogels-design challenges and limitations. Pharmaceuticals. 2022;15:371. 35337169 10.3390/ph15030371 PMC8948902

[2] Mathew AP, Uthaman S, Cho KH, Cho CS, Park IK. Injectable hydrogels for delivering biotherapeutic molecules. Int J Biol Macromol. 2018;110:17–29. 29169942 10.1016/j.ijbiomac.2017.11.113

[3] Øvrebø Ø, et al. Design and clinical application of injectable hydrogels for musculoskeletal therapy. Bioeng Transl Med. 2022. 10.1002/btm2.10295. 35600661 10.1002/btm2.10295 PMC9115710

[4] Hosseinzadeh B, Ahmadi M. Degradable hydrogels: design mechanisms and versatile applications. Mater Today Sustain. 2023;23:100468.

[5] Zöller K, To D, Bernkop-Schnürch A. Biomedical applications of functional hydrogels: innovative developments, relevant clinical trials and advanced products. Biomaterials. 2025;312:122718. 39084097 10.1016/j.biomaterials.2024.122718

[6] Xu F, et al. Hydrogels for tissue engineering: addressing key design needs toward clinical translation. Front Bioeng Biotechnol. 2022;10:849831. 35600900 10.3389/fbioe.2022.849831 PMC9119391

[7] Domínguez-Oliva A, et al. The importance of animal models in biomedical research: current insights and applications. Animals. 2023;13:1223. 37048478 10.3390/ani13071223 PMC10093480

[8] Gao Y, et al. Ionic liquid-based gels for biomedical applications. Chem Eng J. 2023;452:139248.

[9] An S, et al. A miR-activated hydrogel for the delivery of a pro-chondrogenic microRNA-221 inhibitor as a minimally invasive therapeutic approach for articular cartilage repair. Materials today Bio. 2024;30:101382. 39759843 10.1016/j.mtbio.2024.101382 PMC11699623

[10] Shi Y, et al. Injectable doxorubicin-loaded hyaluronic acid-based hydrogel for locoregional therapy and inhibiting metastasis of breast cancer. Colloids Surf B. 2025;247:114433. 10.1016/j.colsurfb.2024.114433 39647423

[11] Gao H, et al. Injectable DAT-ALG hydrogel mitigates senescence of loaded DPMSCs and boosts healing of perianal fistulas in Crohn’s disease. ACS Biomater Sci Eng. 2025. 10.1021/ACSBIOMATERIALS.4C02043. 39804997 10.1021/acsbiomaterials.4c02043

[12] Yun J, Woo HT, Lee S, Cha HJ. Visible light-induced simultaneous bioactive amorphous calcium phosphate mineralization and in situ crosslinking of coacervate-based injectable underwater adhesive hydrogels for enhanced bone regeneration. Biomaterials. 2025;315:122948. 39522352 10.1016/j.biomaterials.2024.122948

[13] Reis-Prado AHdos, et al. Injectable thermosensitive antibiotic-laden chitosan hydrogel for regenerative endodontics. Bioact Mater. 2025;46:406–22. 39850022 10.1016/j.bioactmat.2024.12.026 PMC11754974

[14] Wang Z, et al. Accelerating repair of infected bone defects through post-reinforced injectable hydrogel mediated antibacterial/immunoregulatory microenvironment at bone-hydrogel interface. Carbohydr Polym. 2025;351:123082. 39779005 10.1016/j.carbpol.2024.123082

[15] Qian Z, Qi S, Yuan W. Injectable, self-healing and phase change nanocomposite gels loaded with two nanotherapeutic agents for mild-temperature, precise and synergistic photothermal-thermodynamic tumor therapy. J Colloid Interface Sci. 2025;683:877–89. 39752936 10.1016/j.jcis.2024.12.235

[16] Liu Y, Guo C, Wang Y, Kong QQ. Application of an injectable thermosensitive hydrogel drug delivery system for degenerated intervertebral disc regeneration. Biomacromolecules. 2025;26:209–21. 39670521 10.1021/acs.biomac.4c00965

[17] Delgado JF, et al. In vivo imaging and pharmacokinetics of percutaneously injected ultrasound and X-ray imageable thermosensitive hydrogel loaded with doxorubicin versus free drug in swine. PLoS One. 2024;19:e0310345. 39700200 10.1371/journal.pone.0310345 PMC11658602

[18] Sangboonruang S, et al. Multifunctional poloxamer-based thermo-responsive hydrogel loaded with human lactoferricin niosomes: In vitro study on anti-bacterial activity, accelerate wound healing, and anti-inflammation. International Journal of Pharmaceutics: X. 2024;8:100291. 39493006 10.1016/j.ijpx.2024.100291 PMC11530604

[19] Ugianskiene A, Juhl CS, Glavind K. Role of 3D ultrasound in the treatment of stress urinary incontinence with polyacrylamide hydrogel (Bulkamid®) - a pilot study. Eur J Obstet Gynecol Reprod Biol. 2025;305:218–22. 39708477 10.1016/j.ejogrb.2024.11.041

[20] Zhu F, et al. Photo-crosslinking methacrylated-amylopectin/polyacrylamide hydrogels loading curcumin for applications as degradable, injectable, and antibacterial wound dressings. Int J Biol Macromol. 2024;278:134692. 39154693 10.1016/j.ijbiomac.2024.134692

[21] Ge T, et al. Synthesis and characterization of a novel photothermal hydrogel composite with combined osteogenic and antibacterial activities. Biomed Mater. 2025;20:015037. 10.1088/1748-605X/ada2ce 39715586

[22] Xie Y, et al. Injectable self-healing alginate/PEG hydrogels cross-linked via thiol-Michael addition bonds for hemostasis and wound healing. Carbohydr Polym. 2025;348:122864. 39562129 10.1016/j.carbpol.2024.122864

[23] Ma C, et al. Carboxymethyl chitosan/polyacrylamide double network hydrogels based on hydrogen bond cross-linking as potential wound dressings for skin repair. Int J Biol Macromol. 2024;280:135735. 39293622 10.1016/j.ijbiomac.2024.135735

[24] Pan L, et al. Novel hybrid system based on carboxymethyl chitosan hydrogel encapsulating drug loaded nanoparticles for prolonged release of Vancomycin in the treatment of bacterial infection. J Pharm Sci. 2025;25:00006–11. 10.1016/j.xphs.2025.01.012 39827915

[25] Huang J, et al. An injectable hyaluronic acid/lithium calcium silicate soft tissue filler with vascularization and collagen regeneration. Bioactive materials. 2024;44:256–68. 39507373 10.1016/j.bioactmat.2024.10.014 PMC11539074

[26] Lin C, et al. An injectable in situ-forming hydrogel with self-activating genipin-chitosan (GpCS) cross-linking and an O2/Ca2+ self-supplying capability for wound healing and rapid hemostasis. Carbohydr Polym. 2025. 10.1016/j.carbpol.2024.123051. 39778990 10.1016/j.carbpol.2024.123051

[27] Han C, Zhang H, Wu Y, He X, Chen X. Dual-crosslinked hyaluronan hydrogels with rapid gelation and high injectability for stem cell protection. Sci Rep. 2020. 10.1038/s41598-020-71462-4. 32929113 10.1038/s41598-020-71462-4 PMC7490415

[28] Datta D, et al. Stimuli-responsive self-healing ionic gels: a promising approach for dermal and tissue engineering applications. ACS Biomater Sci Eng. 2025. 10.1021/acsbiomaterials.4c02264. 39999055 10.1021/acsbiomaterials.4c02264 PMC11897956

[29] Raeisi A, Farjadian F. Commercial hydrogel product for drug delivery based on route of administration. Front Chem. 2024;12:1336717. 38476651 10.3389/fchem.2024.1336717 PMC10927762

[30] Sarvepalli S, Kandagatla HP. Injectable hydrogel formulations: design, synthesis and applications for subcutaneous delivery – a comprehensive review. Eur Polym J. 2025;222:113610.

[31] Lou H, Feng M, Hageman MJ. Advanced formulations/drug delivery systems for subcutaneous delivery of protein-based biotherapeutics. J Pharm Sci. 2022;111:2968–82. 36058255 10.1016/j.xphs.2022.08.036

[32] Turner MR, Balu-Iyer SV. Challenges and opportunities for the subcutaneous delivery of therapeutic proteins. J Pharm Sci. 2018;107:1247–60. 29336981 10.1016/j.xphs.2018.01.007 PMC5915922

[33] Coulter SM, et al. In situ forming, enzyme-responsive peptoid-peptide hydrogels: an advanced long-acting injectable drug delivery system. J Am Chem Soc. 2024;146:21401–16. 38922296 10.1021/jacs.4c03751 PMC11311241

[34] Zhao C, Sheng C, Zhou C. Fast gelation of poly (ionic liquid)-based injectable antibacterial hydrogels. Gels. 2022;8:52. 35049587 10.3390/gels8010052 PMC8775204

[35] McCartan A, Mackay J, Curran D, Mrsny RJ. Modelling intramuscular drug fate *in vitro* with tissue-relevant biomimetic hydrogels. International Journal of Pharmaceutics: X. 2022;4:100125. 36065415 10.1016/j.ijpx.2022.100125 PMC9440386

[36] Lawal I, de Castro Araujo Valente D, Khusnatdinov E, Elliott B, Carruth B, Penttila C, Marston J. Effect of orientation angle for needle-free jet injection. Int J Pharm. 2024;664:124612. 10.1016/j.ijpharm.2024.124612 39179006

[37] Zhao T, Wei Z, Zhu W, Weng X. Recent developments and current applications of hydrogels in osteoarthritis. Bioengineering. 2022;9:132. 35447692 10.3390/bioengineering9040132 PMC9024926

[38] Atwal A, Dale TP, Snow M, Forsyth NR, Davoodi P. Injectable hydrogels: an emerging therapeutic strategy for cartilage regeneration. Adv Colloid Interface Sci. 2023;321:103030. 37907031 10.1016/j.cis.2023.103030

[39] Liu W, et al. Intra-articular injectable hydroxypropyl chitin/hyaluronic acid hydrogel as bio-lubricant to attenuate osteoarthritis progression. Mater Des. 2022;217:110579.

[40] Storozhylova N, et al. An in situ hyaluronic acid-fibrin hydrogel containing drug-loaded nanocapsules for intra-articular treatment of inflammatory joint diseases. Regen Eng Transl Med. 2020;6:201–16.

[41] Rai MF, Pham CT. Intra-articular drug delivery systems for joint diseases. Curr Opin Pharmacol. 2018;40:67–73. 29625332 10.1016/j.coph.2018.03.013 PMC6015522

[42] Kalairaj MS, Pradhan R, Saleem W, Smith MM, Gaharwar AK. Intra-articular injectable biomaterials for cartilage repair and regeneration. Adv Healthc Mater. 2024;13:e2303794. 38324655 10.1002/adhm.202303794 PMC11468459

[43] Humphries, D., Baria, M. & Fitzpatrick, J. Severe acute localized reactions after intra-articular hyaluronic acid injections: a narrative review and physician’s guide to incidence, prevention, and management of these adverse reactions. *Journal of Cartilage & Joint Preservation* 100187 (2024) 10.1016/J.JCJP.2024.100187.

[44] García-Couce J, et al. Thermosensitive injectable hydrogels for intra-articular delivery of Etanercept for the treatment of osteoarthritis. Gels. 2022;8:488. 36005089 10.3390/gels8080488 PMC9407145

[45] García-Couce J, et al. Chitosan/Pluronic F127 thermosensitive hydrogel as an injectable dexamethasone delivery carrier. Gels. 2022;8:44. 35049579 10.3390/gels8010044 PMC8774693

[46] Jiang Z, Fu Y, Shen H. Development of intratumoral drug delivery based strategies for antitumor therapy. Drug Des Devel Ther. 2024;18:2189–202. 38882051 10.2147/DDDT.S467835 PMC11179649

[47] Zhao J, Wang L, Zhang H, Liao B, Li Y. Progress of research in in situ smart hydrogels for local antitumor therapy: a review. Pharmaceutics. 2022;14:2028. 36297463 10.3390/pharmaceutics14102028 PMC9611441

[48] Marques AC, Costa PC, Velho S, Amaral MH. Injectable poloxamer hydrogels for local cancer therapy. Gels. 2023;9:593. 37504472 10.3390/gels9070593 PMC10379388

[49] Eltahir S, Al homsi R, Jagal J, Ahmed IS, Haider MG. Graphene oxide/chitosan injectable composite hydrogel for controlled release of doxorubicin: an approach for enhanced intratumoral delivery. Nanomaterials. 2022;12:4261. 36500884 10.3390/nano12234261 PMC9736459

[50] Mantooth SM, et al. Characterization of an injectable chitosan hydrogel for the tunable, localized delivery of immunotherapeutics. ACS Biomater Sci Eng. 2024;10:905–20. 38240491 10.1021/acsbiomaterials.3c01580

[51] Vu TT, et al. Injectable and multifunctional hydrogels based on poly(N-acryloyl glycinamide) and alginate derivatives for antitumor drug delivery. ACS Appl Mater Interfaces. 2024;16:15322–35. 38470564 10.1021/acsami.4c00298

[52] Lee C. Development of injectable and biodegradable needle-type starch implant for effective intratumoral drug delivery and distribution. Int J Nanomedicine. 2022;17:4307–19. 36147547 10.2147/IJN.S370194 PMC9488191

[53] Yun WS, et al. Recent studies and progress in the intratumoral administration of nano-sized drug delivery systems. Nanomaterials. 2023;13:2225. 37570543 10.3390/nano13152225 PMC10421122

[54] Mikhail AS, et al. Hydrogel drug delivery systems for minimally invasive local immunotherapy of cancer. Adv Drug Deliv Rev. 2023;202:115083. 37673217 10.1016/j.addr.2023.115083 PMC11616795

[55] Moshikur RM, et al. Ionic liquids with methotrexate moieties as a potential anticancer prodrug: synthesis, characterization and solubility evaluation. J Mol Liq. 2019;278:226–33.

[56] Cooper RC, Wang J, Yang H. Injectable dendrimer hydrogel delivers Melphalan in both conjugated and free forms for Retinoblastoma. Biomacromolecules. 2024;25:5928–37. 39189328 10.1021/acs.biomac.4c00597 PMC11443594

[57] Rafael D, et al. Delivery systems in ocular retinopathies: the promising future of intravitreal hydrogels as sustained-release scaffolds. Pharmaceutics. 2023;15:1484. 37242726 10.3390/pharmaceutics15051484 PMC10220769

[58] Ottonelli I, et al. Optimization of an injectable hydrogel depot system for the controlled release of retinal-targeted hybrid nanoparticles. Pharmaceutics. 2022;15:25. 36678654 10.3390/pharmaceutics15010025 PMC9862926

[59] Wang L, et al. Injectable drug-loaded thermosensitive hydrogel delivery system for protecting retina ganglion cells in traumatic optic neuropathy. Regen Biomater. 2024;11:rbae124. 39569076 10.1093/rb/rbae124 PMC11578600

[60] Duan N, et al. Biomimetic, injectable, and self-healing hydrogels with sustained release of Ranibizumab to treat retinal neovascularization. ACS Appl Mater Interfaces. 2023;15:6371–84. 36700786 10.1021/acsami.2c17626

[61] Gade SS, et al. Injectable Depot Forming Thermoresponsive Hydrogel for Sustained Intrascleral Delivery of Sunitinib Using Hollow Microneedles. J Ocul Pharmacol Ther. 2022;38:433–48. 35914241 10.1089/jop.2022.0016

[62] Shen C, et al. In situ formation of injectable Gelatin Methacryloyl (GelMA) hydrogels for effective intraocular delivery of Triamcinolone Acetonide. Int J Mol Sci. 2023;24:4957. 36902389 10.3390/ijms24054957 PMC10003315

[63] Ilochonwu BC, et al. Hyaluronic acid-PEG-based Diels-Alder in situ forming hydrogels for sustained intraocular delivery of bevacizumab. Biomacromolecules. 2022;23:2914–29. 35735135 10.1021/acs.biomac.2c00383 PMC9277588

[64] Meany EL, et al. Injectable polymer-nanoparticle hydrogel for the sustained intravitreal delivery of bimatoprost. Adv Ther. 2023;6:2200207.

[65] Akulo KA, Adali T, Moyo MTG, Bodamyali T. Intravitreal injectable hydrogels for sustained drug delivery in glaucoma treatment and therapy. Polymers (Basel). 2022;14:2359. 35745935 10.3390/polym14122359 PMC9230531

[66] Truong D, Wu KY, Nguyen L, Tran SD. Advancements in hydrogel technology for ocular drug delivery. Explor BioMat-X. 2024;1:331–52.

[67] Paez JI, Lim KS. An introduction to injectable hydrogels. J Mater Chem B. 2024;12:5571–2. 38832500 10.1039/d4tb90085e

[68] Huzum B, et al. Biocompatibility assessment of biomaterials used in orthopedic devices: an overview (Review). Exp Ther Med. 2021;22:1315. 34630669 10.3892/etm.2021.10750 PMC8461597

[69] Garcia-Garcia A, et al. Biodegradable natural hydrogels for tissue engineering, controlled release, and soil remediation. Polymers. 2024;16:2599. 39339063 10.3390/polym16182599 PMC11435712

[70] Lu P, et al. Harnessing the potential of hydrogels for advanced therapeutic applications: current achievements and future directions. Signal Transduct Target Ther. 2024;9:166. 38945949 10.1038/s41392-024-01852-x PMC11214942

[71] Hu B, Gao J, Lu Y, Wang Y. Applications of degradable hydrogels in novel approaches to disease treatment and new modes of drug delivery. Pharmaceutics. 2023;15:2370. 37896132 10.3390/pharmaceutics15102370 PMC10610366

[72] Dominijanni AJ, Devarasetty M, Forsythe SD, Votanopoulos KI, Soker S. Cell viability assays in three-dimensional hydrogels: a comparative study of accuracy. Tissue Eng Part C Methods. 2021;27:401–10. 34082602 10.1089/ten.tec.2021.0060 PMC8309413

[73] Karami P, Stampoultzis T, Guo Y, Pioletti DP. A guide to preclinical evaluation of hydrogel-based devices for treatment of cartilage lesions. Acta Biomater. 2023;158:12–31. 36638938 10.1016/j.actbio.2023.01.015

[74] Zhang X, Yin Z, Xiang S, Yan H, Tian H. Degradation of polymer materials in the environment and its impact on the health of experimental animals: a review. Polymers. 2024;16:2807. 39408516 10.3390/polym16192807 PMC11478708

[75] Jeong CH, et al. In vitro toxicity assessment of crosslinking agents used in hyaluronic acid dermal filler. Toxicol In Vitro. 2021;70:105034. 33096205 10.1016/j.tiv.2020.105034

[76] Elmowafy EM, Tiboni M, Soliman ME. Biocompatibility, biodegradation and biomedical applications of poly(lactic acid)/poly(lactic-co-glycolic acid) micro and nanoparticles. J Pharm Investig. 2019;49:347–80.

[77] García-García P, et al. Alginate-hydrogel versus alginate-solid system. Efficacy in bone regeneration in osteoporosis. Mater Sci Eng, C. 2020;115:111009. 10.1016/j.msec.2020.111009 32600680

[78] Suzuki T, et al. Peg shedding-rate-dependent blood clearance of PEGylated lipid nanoparticles in mice: faster PEG shedding attenuates anti-PEG IgM production. Int J Pharm. 2020;588:119792. 32827675 10.1016/j.ijpharm.2020.119792

[79] Yang Q, et al. Analysis of pre-existing IgG and IgM antibodies against polyethylene glycol (PEG) in the general population. Anal Chem. 2016;88:11804–12. 27804292 10.1021/acs.analchem.6b03437 PMC6512330

[80] Stavnsbjerg C, et al. Accelerated blood clearance and hypersensitivity by PEGylated liposomes containing TLR agonists. J Control Release. 2022;342:337–44. 34973307 10.1016/j.jconrel.2021.12.033

[81] Shi D, et al. To PEGylate or not to PEGylate: immunological properties of nanomedicine’s most popular component, polyethylene glycol and its alternatives. Adv Drug Deliv Rev. 2022;180:114079. 34902516 10.1016/j.addr.2021.114079 PMC8899923

[82] Ghattas, M. et al. Chitosan immunomodulation: insights into mechanisms of action on immune cells and signaling pathways. *RSC Adv.* **10**, 15(2), 869–909 (2025). 10.1039/d4ra08406c PMC11719903 39802469

[83] Mndlovu H, Kumar P, du Toit LC, Choonara YE. A review of biomaterial degradation assessment approaches employed in the biomedical field. NPJ Mater Degrad. 2024;8:1–19.

[84] Lee, W. et al. Etiology of Delayed Inflammatory Reaction Induced by Hyaluronic Acid Filler. *Arch Plast Surg* **7**, 51(1), 20–26 (2024). 10.1055/a-2184-6554 PMC10901605 38425859

[85] Correa S, et al. Translational applications of hydrogels. Chem Rev. 2021;121:11385–457. 33938724 10.1021/acs.chemrev.0c01177 PMC8461619

[86] Lu Y, et al. Properties of poly (lactic-co-glycolic acid) and progress of poly (lactic-co-glycolic acid)-based biodegradable materials in biomedical research. Pharmaceuticals. 2023;16:454. 36986553 10.3390/ph16030454 PMC10058621

[87] Valipour F, et al. Thermosensitive and biodegradable PCL-based hydrogels: potential scaffolds for cartilage tissue engineering. J Biomater Sci Polym Ed. 2023;34:695–714. 36745508 10.1080/09205063.2022.2088530

[88] Laffleur F, Netsomboon K, Erman L, Partenhauser A. Evaluation of modified hyaluronic acid in terms of rheology, enzymatic degradation and mucoadhesion. Int J Biol Macromol. 2019;123:1204–10. 30465836 10.1016/j.ijbiomac.2018.11.186

[89] Zhou C, et al. Gradual hydrogel degradation for programable repairing full-thickness skin defect wound. Chem Eng J. 2022;450:138200.

[90] Wu S, Huang J, Jing S, Xie H, Zhou S. Biodegradable shape-memory ionogels as green and adaptive wearable electronics toward physical rehabilitation. Adv Funct Mater. 2023;33:2303292.

[91] Ahn J, Ryu J, Song G, Whang M, Kim J. Network structure and enzymatic degradation of chitosan hydrogels determined by crosslinking methods. Carbohydr Polym. 2019;217:160–7. 31079673 10.1016/j.carbpol.2019.04.055

[92] Deng Y, et al. When can ionic liquids be considered readily biodegradable? Biodegradation pathways of pyridinium, pyrrolidinium and ammonium-based ionic liquids. Green Chem. 2015;17:1479–91.

[93] Kim YE, Kim J. Ros-scavenging therapeutic hydrogels for modulation of the inflammatory response. ACS Appl Mater Interfaces. 2022;14:23002–21. 34962774 10.1021/acsami.1c18261

[94] Lueckgen A, et al. Enzymatically-degradable alginate hydrogels promote cell spreading and in vivo tissue infiltration. Biomaterials. 2019;217:119294. 31276949 10.1016/j.biomaterials.2019.119294

[95] Thomas SN, et al. Liquid chromatography-tandem mass spectrometry for clinical diagnostics. Nat Rev Methods Primers. 2022;2:96. 36532107 10.1038/s43586-022-00175-x PMC9735147

[96] Moyo, MTG. et al. Compliant Hemocompatibility Evaluation of Gellan Gum Hybrid Hydrogels for Biomedical Applications. *Gels* **10**, 824 (2024). 10.3390/gels10120824 PMC11675962 39727582

[97] Mishra K, et al. Ionic liquid-based polymer nanocomposites for sensors, energy, biomedicine, and environmental applications: roadmap to the future. Adv Sci. 2022;9:e2202187. 10.1002/advs.202202187 PMC9475560 35853696

[98] Pu M, et al. ROS-responsive hydrogels: from design and additive manufacturing to biomedical applications. Mater Horiz. 2024;11:3721–46. 38894682 10.1039/d4mh00289j

[99] Preobrazhenskiy II, Putlyaev VI. The ability to control swelling and degradation processes of hydrogels based on a mixture of PEGMA/PEGDA monomers. Mendeleev Commun. 2023;33:83–5.

[100] Zhang Q, et al. Research progress of photo-crosslink hydrogels in ophthalmology: a comprehensive review focus on the applications. Mater Today Bio. 2024;26:101082. 38774449 10.1016/j.mtbio.2024.101082 PMC11107262

[101] Alonso JM, Del Olmo JA, Gonzalez RP, Saez-martinez V. Injectable hydrogels: from laboratory to industrialization. Polymers (Basel). 2021;13:1–24. 10.3390/polym13040650 PMC7926321 33671648

[102] Karami P, et al. Cartilage repair: promise of adhesive orthopedic hydrogels. Int J Mol Sci. 2024;25:9984. 39337473 10.3390/ijms25189984 PMC11432485

[103] Catoira MC, González-Payo J, Fusaro L, Ramella M, Boccafoschi F. Natural hydrogels R&D process: technical and regulatory aspects for industrial implementation. J Mater Sci Mater Med. 2020;31:64. 32696261 10.1007/s10856-020-06401-w PMC7374448

[104] Bashir S, et al. Fundamental concepts of hydrogels: synthesis, properties, and their applications. Polymers (Basel). 2020;12:1–60. 10.3390/polym12112702 PMC7697203 33207715

[105] Ali F, et al. Emerging fabrication strategies of hydrogels and its applications. Gels. 2022;8:205. 35448106 10.3390/gels8040205 PMC9024659

[106] Galante R, Pinto TJA, Colaço R, Serro AP. Sterilization of hydrogels for biomedical applications: a review. J Biomed Mater Res B Appl Biomater. 2018;106:2472–92. 29247599 10.1002/jbm.b.34048

[107] Siqueira ECde, et al. Mecanisms of the chemical crosslinking to obtain the hydrogels: Synthesis, conditions of crosslinking and biopharmaceutical applications. Res Soc Dev. 2023;12:e18312943072.

[108] Park KM, Park KD. In situ cross-linkable hydrogels as a dynamic matrix for tissue regenerative medicine. Tissue Eng Regen Med. 2018;15:547–57. 30603578 10.1007/s13770-018-0155-5 PMC6171695

[109] Qiao H, et al. Scale-up construction of stable multifunctional hydrogel interfaces for large-scale purification of complex oil-water emulsions and oil recovery. J Hazard Mater. 2025;482:136552. 39571378 10.1016/j.jhazmat.2024.136552

[110] Yu AC, et al. Scalable manufacturing of biomimetic moldable hydrogels for industrial applications. Proc Natl Acad Sci U S A. 2016;113:14255–60. 27911849 10.1073/pnas.1618156113 PMC5167152

[111] Mandal A, et al. Hydrogels in the clinic. Bioeng Transl Med. 2020;5:e10158. 32440563 10.1002/btm2.10158 PMC7237140

[112] Robinson TE, et al. The Quantification of Injectability by Mechanical Testing. J Vis Exp. 2020;159:13. 10.3791/61417 32478751

[113] Butreddy A, et al. Molecule Injectables: an Industry Perspective on Formulation Development, Process Optimization, Scale-Up Challenges, and Drug Product Quality Attributes. AAPS PharmSciTech. 2020;21:252. 32885357 10.1208/s12249-020-01787-w

[114] Xu K, et al. Storable hydrogel for infant-friendly oral delivery of amoxicillin for the treatment of pneumococcal pneumonia. ACS Appl Mater Interfaces. 2017;9:18440–9. 28513136 10.1021/acsami.7b01462 PMC5465509

[115] Mohseni-Motlagh SF, Dolatabadi R, Baniassadi M, Baghani M. Application of the quality by design concept (QbD) in the development of hydrogel-based drug delivery systems. Polymers (Basel). 2023;15:4407. 38006131 10.3390/polym15224407 PMC10674248

[116] Krivega ES, Kotova SL, Timashev PS, Efremov YM. Mechanical characterization of soft biomaterials: which time and spatial scale to choose? Soft Matter. 2024;20:5095–104. 38888165 10.1039/d4sm00530a

[117] Ning X, et al. Research advances in mechanical properties and applications of dual network hydrogels. Int J Mol Sci. 2022;23:15757. 36555397 10.3390/ijms232415757 PMC9779336

[118] Raghuwanshi VS, Garnier G. Characterisation of hydrogels: linking the nano to the microscale. Adv Colloid Interface Sci. 2019;274:102044. 31677493 10.1016/j.cis.2019.102044

[119] Romischke J, et al. Swelling and mechanical characterization of polyelectrolyte hydrogels as potential synthetic cartilage substitute materials. Gels. 2022;8:296. 35621594 10.3390/gels8050296 PMC9141488

[120] Azeera, M., Vaidevi, S. & Ruckmani, K. Characterization Techniques of Hydrogel and Its Applications. *Polymers and Polymeric Composites* 737–761 (2019) 10.1007/978-3-319-77830-3_25.

[121] Budi HS, et al. Injectable and 3D-printed hydrogels: state-of-the-art platform for bone regeneration in dentistry. Inorg Chem Commun. 2024;161:112026.

[122] Mohan Dodda, J., Ashammakhi, N. & Rotimi Sadiku, E. *Injectable Smart Hydrogels for Biomedical Applications*. *Injectable Smart Hydrogels for Biomedical Applications* (Royal Society of Chemistry, 2024). 10.1039/9781837673070.

[123] Mohaghegh N, et al. Injectable hydrogels for personalized cancer immunotherapies. Acta Biomater. 2023;172:67–91. 37806376 10.1016/j.actbio.2023.10.002

[124] Ran P, et al. Light-triggered theranostic hydrogels for real-time imaging and on-demand photodynamic therapy of skin abscesses. Acta Biomater. 2023;155:292–303. 36435439 10.1016/j.actbio.2022.11.039

